# Effects of congeners of amphetamine on the human heart

**DOI:** 10.1007/s00210-024-02983-2

**Published:** 2024-02-10

**Authors:** Joachim Neumann, Stefan Dhein, Uwe Kirchhefer, Britt Hofmann, Ulrich Gergs

**Affiliations:** 1 Institut für Pharmakologie und Toxikologie, Medizinische Fakultät, Martin-Luther-Universität Halle-Wittenberg, Magdeburger Str. 4, 06112, D-06097 Halle, Germany; 2 Rudolf-Boehm Institut für Pharmakologie und Toxikologie, Universität Leipzig, Härtelstraße 16-18, D-04107 Leipzig, Germany; 3 Universität Münster, Münster, Germany; 4 Cardiac Surgery, Medizinische Fakultät, Martin-Luther-Universität Halle-Wittenberg, D-06097 Halle, Germany

**Keywords:** Congeners, Amphetamine, Human heart

## Abstract

Central stimulatory and hallucinogenic drugs of abuse like amphetamine and most congeners of amphetamine can have cardiac harmful effects. These cardiac side effects can lead to morbidities and death. In this paper, we review current knowledge on the direct and indirect effects of these amphetamine congeners on the mammalian heart—more specifically, the isolated human heart muscle preparation. In detail, we address the question of whether and how these drugs affect cardiac contractility and their mechanisms of action. Based on this information, further research areas are defined, and further research efforts are proposed.

## Introduction

**Fig. 1:**
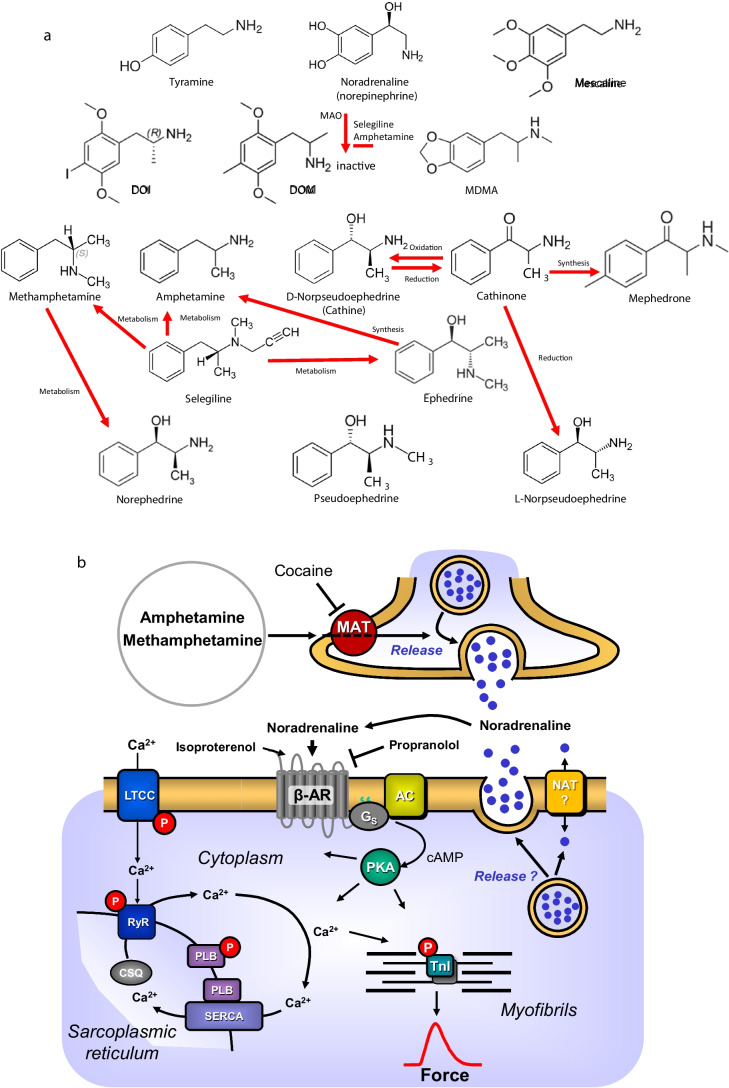
**a** Structural formulae. Depicted are of drugs of interest namely amphetamine (amfetamine), cathine, cathinone, 2,5-dimethoxy-4-iodoamphetamine (DOI), 2,5-dimethoxy-4-methylamphetamine (DOM), ephedrine, 3,4-methylene-dioxymethamphetamine (MDMA), mephedrone, mescaline, methamphetamine (methamphetamine), norephedrine, norpseudoephedrine and pseudoephedrine. For comparison, the structural formulae of noradrenaline, selegiline and tyramine are shown. Arrows indicate on top the inactivation of noradrenaline by mono-oxidases (MAO) and their inhibition by selegiline or amphetamine. Other arrows indicate the interconversion by metabolism or oxidation or reduction. Finally, the arrow labelled with synthesis indicate that in vitro synthesis has been reported. **b** Scheme on possible mechanisms of action of amphetamine or methamphetamine and related compounds. Cocaine inhibits the activity of several different monoamine transporters (MAT). Methamphetamine or related compounds entering cells via cocaine inhibitable transporters might release noradrenaline from nerve terminals or cardiomyocytes. Noradrenaline like isoprenaline (isoproterenol) can activate sarcolemmal β-adrenoceptors. This leads via stimulatory G-proteins (G_s_) and activation of adenylyl cyclases to subsequent production of cAMP. The cAMP activates the cAMP-dependent protein kinase (PKA). PKA increases cardiac force generation and relaxation by increasing the phosphorylation state (P) of the L-type calcium ion channel (LTCC), of phospholamban (PLB) and of the inhibitory subunit of troponin (TnI). Trigger Ca^2+^ initiates release of Ca^2+^ from the sarcoplasmic reticulum via ryanodine receptors (RYR) into the cytosol. There, Ca^2+^ activates myofilaments and this activation leads to increased inotropy. In diastole, Ca^2+^ is taken up into the sarcoplasmic reticulum via a sarcoplasmic reticulum Ca^2+^-ATPase (SERCA), the activity of which is enhanced due to an increased phosphorylation state of PLB. Ca^2+^ enters the cardiomyocyte via the LTCC. Phosphodiesterase 3 and 4 degrades cAMP (this degradation is blocked by cilostamide in man or in mouse by rolipram, respectively). Amphetamine can lead via release of noradrenaline to stimulation of β-adrenoceptors. This will via the delineated pathway, increase the phosphorylation state of PLB. Thence, SERCA pumps per unit of time more Ca^2+^ into the SR. When Ca^2+^ leaks from the SR into the cytosol, then Ca^2+^can be extruded from the cytosol via the electrogenic sarcolemmal sodium/calcium ion exchanger (not shown). Thereby, depolarization can follow. This typically causes atrial fibrillation due to delayed afterdepolarizations (review: Heijman et al. 2014). In this fashion, amphetamine and its congeners may cause cardiac arrhythmias

When we refer subsequently to “drug(s) of interest”, we mean the following drugs we chose here because they are structurally related (Fig. 1a): amphetamine (amfetamine), cathine, cathinone, 2,5-dimethoxy-4-iodoamphetamine (DOI), 2,5-dimethoxy-4-methylamphetamine (DOM), ephedrine, 3,4-methylene-dioxymethamphetamine (MDMA), mephedrone, mescaline, methamphetamine (methamphetamine), norephedrine and pseudoephedrine (Table 1, structural formulae: Fig. 1a). These drugs of interest are related to the lead compound amphetamine (Fig. 1a). Of course, they also show similarities to noradrenaline (Fig. 1a). Hence, the drugs of interest could theoretically act like noradrenaline as agonists at adrenergic receptors. However, this is seldom ever the case. All these drugs, at high plasma levels, may be hallucinogenic agents in humans, most probably via direct or indirect stimulation of 5-HT_2A_-serotonin receptors in the brain (review: Gumpper and Roth 2023).

**Table 1:** Source of the drugs of interest

	Source	Number of chiral atoms	References
Amphetamine	Laboratory	One	Edeleano 1887
Cathine: D-norpseudoephedrine	Plant: Catha edulis	Two	Wolfes 1930, Kalix 1983
Cathinone	Plant: Catha edulis	One	Friebel and Brilla 1963
DOI	Laboratory	One	Shulgin et al. 2011
DOM	Laboratory	One	Snyder et al. 1968
Ephedrine	Plant: Ephedra sinica	Two	Nagai 1897
L-Norpseudoephedrine	Plant: Ephedra sinica	Two	Krizevski et al. 2007
MDMA: 3,4-methylene-dioxymethamphetamine	Laboratory	Two	Köllisch 1912, Merck company 1914
Mephedrone: 4-methyl-methcathinone	Laboratory	One	Sanchez 1929
Mescaline	Plant: Lophophora williamsii	None	Heffter 1894, Lewin 1888, Späth 1919
Methamphetamine	Laboratory	One	Nagai 1897
Norephedrine	Plants: Ephedra sinica, Ephedra vulgaris	Two	Krizevski et al. 2007
Pseudoephedrine	Plants: Catha edulis, Sida cordifolia, Roemeria refracta	Two	Ladenburg and Oelschlägel 1889

The first column lists the drugs of interest. The term “laboratory” means in the second column: this compound is not detected in a plant but is produced by organic chemical synthesis in the laboratory. Naturally occurring drugs are given with a typical name of their plant of origin. In the third column for each drug of interest the number of chiral atoms that is chiral carbon atom in that molecule are given. They range from zero to two. Drugs of different chirality can have different pharmacological properties. Fourth column gives references for each drug in the row.

All of the drugs of interest can have severe side effects. Most are currently illicitly but widely used as “recreational drugs”. Some of these drugs of interest currently undergo a clinical re-evaluation and may be approved for the treatment of psychiatric diseases (e.g. MDMA in Australia: Haridy 2023). Hence, a better knowledge of the cardiovascular effects of these drugs of interest is not only of toxicological relevance (treatment of recreational users), but potentially clinically useful and will, therefore, be presented here.

One can find metabolical interconversion in the human body (also vide infra), or at least interconversion in the chemical laboratory between the drugs of interest (Fig. 1a); amphetamine is formed as a metabolite of methamphetamine in the human body by demethylation. Hence, we might regard amphetamine as an active metabolite of methamphetamine. Modification of the phenyl group of amphetamine leads to 2,5-dimethoxy-4-iodoamphetamine (DOI) and 2,5-dimethoxy-4-methylamphetamine (DOM). Methamphetamine can be chemically produced from ephedrine. Therefore, ephedrine is a controlled substance. Ephedrine has two chiral carbon atoms, like its demethylated derivative, norephedrine (Table 1). Furthermore, the demethylation of pseudoephedrine produces cathine. Oxidation of cathine brings us to cathinone. Methylation of the side chains of noradrenaline (Fig. 1a) leads to MDMA. Side chain modifications of MDMA lead to mescaline (Fig. 1a).

While most derivatives of noradrenaline are also centrally stimulant drugs via brain 5-HT_2A_-serotonin receptors or brain adrenergic receptors, there are exceptions to this rule: tyramine (2-(4-hydroxyphenyl)ethylamine (Fig. 1a) formed from tyrosine) is a well-known indirect sympathomimetic compound (in humans: e.g. Iepsen et al. 2018) but, to the best of our knowledge, not a central stimulant agent. On the one hand, tyramine is rapidly metabolised to inactive compounds by monoamine oxidases (MAO) in the body. On the other hand, tyramine does not readily pass the blood–brain barrier (Ghose 1984). For these reasons, it is likely that no relevant concentrations of tyramine reach the brain. Therefore, tyramine has no psychedelic effect. In contrast to noradrenaline or tyramine, the drugs of interest, that do not contain hydroxyl groups in their benzene ring, are poor substrates to intestinal MAO and, hence, are not relevantly altered in the gastrointestinal tract. Therefore, all drugs of interest can be applied through the mouth. Moreover, they readily pass the blood–brain barrier and hence can exert their hallucinogenic effects.

Besides their structural classification (Fig. 1a), another classification of drugs of interest might reside in their origins (Table 1). Several compounds were only produced in the chemistry laboratory and surprisingly have not (yet) been found in nature; here, methamphetamine, MDMA and amphetamine could be placed (Table 1). The other drugs of interest occur in plants, such as cathine and cathinone in *Catha edulis* (khat) leaves or pseudoephedrine, ephedrine and norephedrine (Table 1). Mescaline is produced in a cactus called “peyote” by the autochthonous population.

These hallucinogenic drugs of interest are located on continents around the globe: ephedrine in Asia (China), cathine in the Horn of Africa, or the southern Arabian Peninsula, whereas peyote is found in the Americas (Table 1).

Amphetamine (vide infra) typically acts as an indirect sympathomimetic compound. Indirect sympathomimetic compounds are drugs that release noradrenaline from storage sites in cells (Fig. 1b). Typically, these indirect sympathomimetic drugs do not act by themselves as agonists at adrenoceptors (Goodman and Gilman 2023). Further understanding of the mechanism(s) of indirect sympathomimetic drugs like amphetamine can be obtained from the use of cocaine. Cocaine inhibits the reuptake of noradrenaline into neuronal cells. Therefore, cocaine increases the effects of noradrenaline on the heart by increasing the concentrations of noradrenaline near interstitial sarcolemmal β-adrenoceptors in the heart (Fig. 1b). Thus, while cocaine increases the potency of noradrenaline to exert a positive inotropic effect, it can nullify the positive inotropic effects of amphetamine in the animal heart.

As illustrated in Fig. 1b, cocaine inhibits the uptake of amphetamine-like compounds into cells, namely neuronal cells. This leads to lower concentrations of amphetamine-like compounds in the cytosol; thus, amphetamine-like compounds cannot compete with noradrenaline for transport enzymes within the cell. In the absence of cocaine, amphetamine-like compounds induce the release of noradrenaline from storage vesicles within cells and thus increase the concentration of noradrenaline in the cytosol. Such high concentrations of noradrenaline in the cytosol drive the extrusion of noradrenaline from cells into the extracellular space. Having reached the extracellular space, noradrenaline can access the sarcolemmal adrenoceptors on cardiomyocytes in the myocardium, increasing the function of the heart.

### Amphetamine

As alluded to above, amphetamine (short for alpha-methylphenethylamine, or β-amino-propyl-benzene, trade name: Benzedrine®, street name: speed) is a methylated derivative of α-phenylethylamine (Fig. 1a). Amphetamine was synthesised in an extensive series of chemically related compounds formed by the same chemical reaction using different reaction partners (Edeleano 1887, Table 1). This work was driven by pure chemical curiosity (a doctoral thesis in chemistry); no application in humans was intended, and no biological studies of amphetamines were conducted (Edeleano 1887).

In the 1930s, amphetamine was used to treat asthma (Morelli and Tognotti 2021). This finding was probably driven by the desire in the United States of America (USA) to find a cheap substitute for ephedrine (imported in the 1930s from China). In the 1930s, ephedrine (vide intra) was used in asthmatics based on reports in traditional Chinese medicine (Lee 2011). Amphetamine was not only orally applied but was also popular in an inhaler to treat asthma until newer drugs entered the market (Morelli and Tognotti 2021). Also, in the 1930s, amphetamine was observed (conceivably in asthmatics treated with amphetamine) to reduce hunger. Thus, amphetamine was tried successfully to treat obesity and binge eating (Morelli and Tognotti 2021, Table 5). Likewise, in the 1930s, amphetamine was found to help treat children with attention-deficit and hyperactivity disorders (ADHD) (Morelli and Tognotti 2021). These indications are still valid today.

Amphetamine was marketed without prescription by physicians until 1959, which might explain the wide use of amphetamine in the USA (Morelli and Tognotti, 2021). Amphetamine became a “recreational” drug when its centrally stimulatory actions became widely known in the 1930s, probably in asthmatics. These centrally stimulatory actions of amphetamine include hallucinations (Shoptaw et al. 2009, Table 4). Mainly in the USA, amphetamine was used by athletes for doping purposes. In German athletes, methamphetamine was more prevalent because it could be produced cheaply (Morelli and Tognotti 2021, vide infra). The North American medal-winning athletes in the 1936 Berlin Olympic Games were notorious for amphetamine misuse to improve their cardiovascular function (Morelli and Tognotti 2021). The high number of Olympic medals for the US team in 1936 may have resulted from doping with amphetamine (Morelli and Tognotti 2021). Even today, amphetamine is used as a doping agent in athletes. As a result, amphetamine was added to the World Anti-Doping Agency’s (WADA) doping list (Table 5, Docherty 2008). The successes of the US team in 1936 reportedly motivated German chemists and physicians to develop a cheap synthesis for methamphetamine, the competing German doping drug (vide infra, Ohler 2016, Morelli and Tognotti 2021).

The use of methamphetamine after 1936 became widespread, first among athletes and then among German soldiers (Ohler 2016, Morelli and Tognotti 2021, vide infra). In contrast, in the Armed Forces of the US during the Second World War (1941–1945), amphetamine was routinely given to improve cardiovascular performance, improve mood and increase the alertness of soldiers (Morelli and Tognotti 2021). In 1942, Allied soldiers in Northern Africa benefited from at least half a million tablets of amphetamine (Ohler 2016). This was equivalent to a massive Phase IV drug trial. Amphetamine is still being used by soldiers worldwide (Eliyahu et al. 2007).

In amphetamine, several action mechanisms are observed in the central nervous system, some of which may also be operational in the heart. Amphetamine released (with increasing potency) serotonin (5-HT), dopamine and noradrenaline from rat brain preparations (Rothman et al. 2003). Thus, amphetamine is thought to act indirectly at the central and cardiac adrenoceptors via noradrenaline or dopamine or 5-HT as the intermediates (Fig. 1b, Liles et al. 2007, Goodman and Gilman 2023). The central release of 5-HT by amphetamine probably explains why amphetamine can stimulate central 5-HT_2A_-serotonin receptors. Stimulation of these 5-HT_2A_-serotonin receptors by amphetamine or by the other “drugs of interest” explains why amphetamine and its congeners can induce hallucinations (Shoptaw et al. 2009).

In the hearts of living animals or their isolated cardiac preparations after pre-treatment with reserpine, the effects of amphetamine were missing (e.g. Liles et al. 2007). Reserpine probably removed noradrenaline from cardiac storage sites. In these experiments, it is likely that reserpine inhibited the activity of the vesicular monoamine transporter (VMAT2). Thus, reserpine inhibits the storage of noradrenaline in nerve vesicles. When these noradrenaline stores are no longer filled with endogenous noradrenaline from the prolonged treatment of living animals with reserpine, subsequent amphetamine is probably unable to release noradrenaline. Thus, amphetamine loses its cardiac effects, including its positive inotropic effect.

Additional mechanisms of action have been noted for amphetamine. In the brain, amphetamine inhibits the transporters of dopamine, 5-HT, noradrenaline, VMAT2 and MAO (Table 7). Amphetamine increases the concentration of dopamine, 5-HT and noradrenaline in the central nervous system (review: Faraone 2018). Hence, one would predict that similar mechanisms should prevail in the heart. Amphetamine is expected to potentiate the inotropic, chronotropic and pro-arrhythmic effects of noradrenaline and dopamine on β-adrenoceptors in the heart. With similar mechanisms as in the brain, amphetamine might release dopamine from cardiac storage sites. This released cardiac dopamine can stimulate α-, β-adrenergic and conceivably D_1_-dopamine receptors to increase the cardiac force of contraction (review: Neumann et al. 2023a). It is currently unclear to what extent the cardiac effects of amphetamine are dopamine- or dopamine-receptor-mediated. In contrast, amphetamine failed to increase the myocardial 3´,5´-cyclic adenosine monophosphate (cAMP) content in minced rat hearts (Hull et al. 1993).

Amphetamine exerts a wide range of cardiovascular effects. For example, amphetamine has vasoconstrictory effects. These vasoconstrictory effects of amphetamine are attenuated by pre-treatment of animals using reserpine (vide supra). They are lacking in mice with general knockout of dopamine β-hydroxylase (the pacemaker enzyme for synthesis of noradrenaline), where cardiac and adrenal and central noradrenaline levels were significantly reduced (Liles et al. 2007). These studies suggest that amphetamine is an indirect sympathomimetic agent, at least in animals (Liles et al. 2007). Surprisingly, in isolated left atrial preparations from guinea pigs, 148 μM of amphetamine failed to alter the force of contraction (Khoyi et al. 1978). This might mean that there are few noradrenaline stores in the guinea pig atrium. In contrast, amphetamine quickly increased the force of contraction in a time- and concentration-dependent way in mouse left atrial preparations and in isolated human right atrial preparations (Hußler et al. 2023, Neumann et al. 2023d).

These positive inotropic effects of amphetamine in human atrial preparations were significantly attenuated by 10 μM cocaine, abrogated by 10 μM propranolol and potentiated by phosphodiesterase inhibitors at least in mouse-isolated cardiac preparations (Hußler et al. 2023, Fig. 1b). The positive inotropic effects and lusitropic effects of amphetamine in the isolated human right atrium were accompanied by increases in the phosphorylation state of troponin (Hußler et al. 2023). These direct cardiac effects of amphetamine are probably relevant. The therapeutic dosage of amphetamine leads to plasma concentrations of amphetamine of around 0.7 μM (Table 3). Higher concentrations were measured in the intoxicated patients (Table 3).

In the brain, but conceivably also in the human heart, amphetamine might enhance concentrations of acetylcholine (Verhheijen et al. 2009) or serotonin (Coleman et al. 2019; Kristensen et al. 2011). Serotonin via 5-HT_4_ serotonin receptors in the human heart would act like the stimulation of β-adrenoceptors (Neumann et al. 2023a). Specifically, serotonin increases the force of contraction and beating rate and can elicit arrhythmias in the human atrium (Neumann et al. 2023a). Amphetamine can lead via release of noradrenaline to stimulation of β-adrenoceptors. Activated β-adrenoceptors lead to an increase in the phosphorylation state of phospholamban (Fig. 1b). Thence, more Ca^2+^ is stored in the sarcoplasmic reticulum (SR). When Ca^2+^ leaks from the SR into the cytosol, then Ca^2+^can be extruded from the cytosol via an electrogenic sarcolemmal sodium/calcium ion exchanger. Three molecules of Na^+^ enter the cardiomyocytes while one mole of Ca^2+^T exits the cell. Hence, the cell potential changes to the positive and this can lead with time to cell depolarization. This can cause fatal cardiac arrhythmia (review: Heijman et al. 2014). In this fashion, amphetamine but also its congeners, that also release noradrenaline from cardiac stores, may cause cardiac arrhythmias.

The release of noradrenaline, typically is thought to occur in nerve cells in the heart (Fig. 1b). However, it has been known now for a while, that cardiomyocytes contain noradrenaline (Neumann 2006). This noradrenaline can be formed in the cardiomyocytes but may in part also derive from other cells and may then enter the cardiomyocytes (Neumann 2006). Hence, it is not unreasonable to assume that amphetamine or its congeners might, to some extent, release noradrenaline from cardiomyocytes. But this speculation needs to be tested by adding, e.g. amphetamine to cultured cardiomyocytes and then measure the concentration of noradrenaline in the supernatant of the cultured cells. Moreover, one would predict that amphetamine would increase the phosphorylation state of phospholamban in cultured cardiomyocytes. This phosphorylation should be inhibited by cocaine and by propranolol as they independently would impair this predicted pathway in the cardiomyocytes (Fig. 1b).

The opposite functions are expected from acetylcholine, negative inotropic effect and a negative chronotropic effect (Goodman and Gilman 2023). However, amphetamine increases and does not decrease the force of contraction in isolated human atrial preparations, which are very sensitive to acetylcholine (Hußler et al. 2023). However, one could study in the future whether the positive inotropic effects of amphetamine are more significant in the presence of atropine to block muscarinic receptors in isolated human atrial preparations. This would be circumstantial evidence for a release of acetylcholine by amphetamine in the heart.

One could argue that more than a single application, the chronic application of amphetamine in experimental animals is more relevant clinically. This is because amphetamine is addictive in humans, and thus, amphetamine is applied many times to treat this craving. Thus, the repeated application of amphetamine has often been investigated. When this repeated or chronic application of amphetamine is investigated in experimental animals, the expression of many genes on mRNA and protein levels changed (Brain: Sokolov et al. 2003). Thus, amphetamine cannot only induce short-term effects in the heart (e.g. via the release of catecholamine) but also long-lasting changes in protein expression. These changes in protein expression, if they occur in proteins relevant to cardiac function, should also alter the positive inotropic and chronotropic effects of amphetamine in the heart. However, such contractile data have apparently not yet been reported.

Recreational drugs, like amphetamine, can lead to intoxication and death. One reason for a fatal course of intoxication with amphetamine might lie in cardiac arrhythmias. One manifestation of cardiac arrhythmias is tachycardia (Tables 4 and 6). Amphetamine can cause coronary constriction due to noradrenaline acting on the α-adrenoceptor in the vessel wall. This reduces cardiac blood flow and might cause cardiac arrhythmias. For instance, 2 mg/l for amphetamine in intoxication (15 μM, Table 3, Uekusa et al. 2013).

The question may arise as to whether propranolol could be used to treat intoxication with amphetamine. There are case reports that the release of noradrenaline by propranolol in patients intoxicated with amphetamine is unopposed at the α-adrenoceptors and leads to hypertension and stroke (Spiller et al. 2013). Hence, the use of a blocker like labetalol at both adrenergic receptors or the additional use of an antagonist like prazosin at the α-adrenoceptors might be considered.

Interestingly, amphetamine is a metabolite of selegiline in patients (Fig. 1a, Shin 1997). Selegiline is used to inhibit MAO in the treatment of Morbus Parkinson and depression (Shin 1997). The antidepressant effect of selegiline might stem thus in part from its active metabolite, amphetamine. Moreover, the cardiovascular side effects of selegiline might also be caused by amphetamine.

Currently, amphetamine is sometimes clinically used to treat obesity, narcolepsy and attention-deficit disorder (ADD) (Table 2). More often, amphetamine is used as a drug of abuse and as a stimulant for recreational purposes. There have been 136 studies of amphetamine in clinical trials (Table 2). These studies make it obvious that research is ongoing on other indications, such as depression for amphetamine (Table 2). Hence, one cannot rule out the possibility that new indications for amphetamine might be found, which makes it even more important to know the cardiovascular effects of amphetamine.

**Table 2:** Clinical studies and tested indications of the drugs of interest

	Clinical Studies in ClinicalTrials.gov/publications in PubMed	Some tested indications for the drugs in ClinicalTrials.gov	Further indications
Amphetamine	136/50,823	Depression, treatment of amphetamine dependence	Attention-deficit/hyperactivity disorder: Heal et al. 2013
Cathine: D-norpseudoephedrine	None/283	None	Obesity: Hauner et al. 2017
Cathinone	None/1812	None	aphrodisiac: Bentur et al. 2008
DOI	None/970	None	Basic research, anti-inflammatory (Flanagan and Nichols 2018)
DOM	None/380	None	basic research
Ephedrine	765/6969	Hypotension, hypothermia, obesity, preeclampsia	Narcolepsia: Gad et al. 2021
L-Norpseudoephedrine	None/233	None	Binge eating: Nencini et al. 1996
MDMA: 3,4-methylene-dioxymethamphetamine	136/5053	Post-traumatic stress disorder, social anxiety, treatment of MDMA dependence	Autism: Pitts et al. 2018
Mephedrone: 4-methyl-methcathinone	2/695	Pharmacokinetics and interaction with alcohol	Basic research
Mescaline	6/1332	Mechanism of action of mescaline	Alcoholism: Vamvakopoulou et al. 2023
Methamphetamine	437/16,685	Treatment of methamphetamine dependence	Attention-deficit disorders, obesity: Abbruscato and Trippier 2018
Norephedrine	56/3700	Keratitis, cough, respiratory tract infection	Obesity: Ryan 1996
Pseudoephedrine	774/995	Rhinitis, “common cold”, hypotension, barotrauma	Neurogenic shock: Wood et al. 2014

In the first column the drugs of interest are listed. In the second column, clinical studies indicate the number of studies for this drug of interest found as of 11/4/23 at the website “ClinicalTrials.gov”., PubMed means how many publications for this compound are found as of 11/4/23 in “PubMed (nih.gov)”. There were no listed publications for L-norpseudoephedrine in “PubMed”; therefore, we give here the number of publications for norpseudoephedrine which may contain publications for L-norpseudoephedrine. These numbers are given as an objective parameter for the worldwide interest in the drugs of interest in the clinic but also in general. There seems to be a positive correlation between these parameters. On the other hand, there are exceptions namely if one looks at norephedrine

### Cathine

Cathine was first isolated from khat leaves by Beitter (1901) and falls into the group of phenyl-hydroxy-propanolamines. Cathine is also called β-hydroxy-amphetamine (Wolfes 1930, Table 1). In other words, cathine might be regarded as an amphetamine derivative (Fig. 1a). Phenyl-hydroxy-propanolamines possess two asymmetric carbon atoms (like ephedrine, Table 1) numbered 1 and 2. Hence, four optical isomers can exist: 1S,2S-, 1S,2R-, 1R,2S- and 1R,2R–phenyl-hydroxy-propanolamine (Fig. 1a). For historical reasons, their effects are usually published under vernacular names. For instance, 1S,2S-phenyl-hydroxy-propanolamine is also known as 1S,2S-norpseudoephedrine, or D-norpseudoephedrine or (+)-norpseudoephedrine or pseudo-nor-ephedrine or cathine (Fig. 1a). The enantiomer of cathine is thus 1R,2R-phenyl-hydroxy-propanolamine and is also called L-norpseudoephedrine or (-)-norpseudoephedrine. 1R,2S-phenyl-hydroxy-propanolamine is L-norephedrine or (-) norephedrine. Thus, the 1R,2S-phenyl-hydroxy-propanolamine or D-norephedrine is the enantiomer of L-norephedrine or (+)-norephedrine. 1S,2R- and 1R,2R-phenyl-hydroxy-propanolamines are not synthesised by plants and, to our knowledge, do not occur in nature.

As mentioned above, the leaves of the Catha edulis tree contain cathine. Leaves of Catha edulis (khat) are usually chewed in ceremonies connected to social gatherings but also in religious ceremonies to induce hallucinations in an area of the globe, which is also thought to be the home of coffee (Coffea Arabica, Rätsch 2018). Cathine has been noted to cause hallucinations (Rieger 1981, Silva et al. 2022). The mysteries in the Greek Delphic Oracle have been claimed to result from inhalation of fumes containing cathine (Rätsch 2018). Khat chewing dates much farther back than coffee drinking (Kalix et al. 1983). In addition, tea from khat is consumed, and khat is also smoked in Arabia (Rätsch 2018). There is some claim that khat has aphrodisiac effects. Moreover, some data indicate that, in animal studies, protracted khat consumption increases spermatogenesis (discussed in Adeoya-Osiguwa and Fraser 2005).

Cathine is also consumed in Europe. At least in central London (Great Britain), in a nightlife district, cathine was detected in public urinals as often as cocaine (Archer et al. 2013). The presence of cathine was not from khat, but probably (Archer et al. 2013) from over-the-counter nasal decongestants containing pseudoephedrine (vide infra). In vitro, cathine inhibited the activity of CYP2D6 and CYP3A4 (Lim et al. 2022). In humans, khat consumption reduces the activity of CYP2D6 (Silva et al. 2022). Hence, increased levels of cathine are expected in humans taking khat and inhibitors of CYP2D6 (Silva et al. 2022). In rats and mice, cathine had anorectic effects, reducing their total body eight (e.g. Blosser et al. 1987, Arch et al. 1982).

It has been claimed that the drug Nepenthes mentioned by the Greek poet Homer (author of Iliad and Odyssey) contained cathine, that cathine was used in Ancient Egypt, or that the Greek conqueror Alexander the Great gave cathine-containing herbs to his soldiers to endure marching long distances (Rätsch 2018). Fittingly, cathine is now found on the WADA doping list (Docherty 2008, WADA, Table 5).

Cathine is formed by reducing cathinone (Fig. 1a) in plants. Cathine is found naturally in several plants, such as Catha edulis (also called khat: Kalix 1983), Ephedra sinica or Ephedra equiseta (Table 1). Catha edulis contains the highest concentration of cathine compared to other plants. Catha edulis is found in the Arabic peninsula (Yemen, Aden) and the Horn of Africa (Ethiopia, Somalia, Kenya) but also in Tanzania and Uganda (Rätsch 2018). Catha edulis is also cultivated in Afghanistan, Israel, Madagascar and Kazakhstan (Rätsch 2018).

Cathine can release dopamine and noradrenaline, but not serotonin, from rat brain preparations (Rothman et al. 2003, Table 7). Cathine is transported through membranes by a protein called OCT2 (Jensen et al. 2021). If this transporter, which also occurs in the heart, were blocked, the effect of cathine within the cells would possibly vanish. Moreover, cathine inhibits the activity of dopamine transporting proteins (DAT) and noradrenaline transporting proteins (NET) in cell cultures (Jensen et al. 2021, Table 8). Hence, cathine should potentiate the effects of dopamine and, more likely, noradrenaline in the heart. However, cathine can inhibit serotonin transporters (Jensen et al. 2021) and thus might potentiate the cardiac effects of serotonin at human 5-HT_4_-serotonin receptors.

There is limited evidence that, at least in mouse sperm, cathine can increase cAMP levels supposedly via β-adrenoceptors (Adeoya-Osiguwa and Fraser 2005). In contrast, cathine failed to increase the myocardial cAMP content in minced rat hearts (Hull et al. 1993). High concentrations of cathine can inhibit the activity of MAO enzymes, another way that noradrenaline levels might be increased in the heart for a prolonged period (Nencini et al. 1984). Moreover, khat extracts behave immunologically differently from cathine extracts. While khat extracts increase the phosphorylation state transcription factors of human leucocyte subsets, cathine usually reduces their phosphorylation state (Berdholdt et al. 2013). As these transcription factors are also phosphorylated in the human heart, it is possible that such changes also occur in frequent users. How this alters cardiac function needs to be studied.

All these effects are explained by the action of cathine, which releases noradrenaline in the body (Table 7). This noradrenaline stimulates brain and peripheral adrenergic receptors.

Khat chewing has predictable side effects. In the mouth, local infection, dysplastic tissue and periodontitis have been reported; this can be explained by vasoconstrictory components of khat. Khat chewing increases blood pressure and heart rate and can lead to heart failure (El-Menyar et al. 2015, Table 4).

Oxidation of cathine (D-norpseudoephedrine) will lead to S-(-)cathinone (Fig. 1a, Table 1, May et al. 1982, vide infra); one from two possible centres of chirality is lost by this reduction of the chiral hydroxy group to a non-chiral keto group. Cathine raises blood pressure in rats (Moya-Huff et al. 1987). Cathine (D-norpseudoephedrine) is present in all drug preparations containing racemic norpseudoephedrine. The therapeutic drug levels of cathine are around 0.45 μM (Table 3). At this concentration, one can detect cardiac effects of cathine in isolated cardiac preparations. In mice, cathine leads to hyperthermia (Arch et al. 1982). In humans, khat leaf consumption leads to elevated body temperature (Toennes et al. 2003). This may be of cardiac relevance, because hyperthermia in humans increases the incidence of arrhythmias (fever arrhythmias).

**Table 3:** “Therapeutic” and toxic concentrations of the drugs of interest

	Therapeutic	Toxic	References
Amphetamine	0.74 μM^2^	15 μM^1^	^1^Uekusa et al. 2013 ^2^Holze et al. 2020
Cathine: D-norpseudoephedrine	0.45 μM		Toennes et al. 2003
Cathinone	0.34 μM^3^, 0.5 μM^1^, 0.7 μM^2^		^1^Brenneisen et al. 1990 ^2^Widler et al. 1994 ^3^Toennes et al. 2003
DOI	n.d.		
DOM	n.d.		
Ephedrine	1.12 μM		Vanakoski et al. 1993
L-Norpseudoephedrine	0.65 μM		Widler et al. 1994
MDMA: 3,4-methylene-dioxymethamphetamine	1.22 μM		Holze et al. 2020
Mephedrone: 4-methyl-methcathinone	0.73 μM	124 μM	Papaseit et al. 2017
Mescaline	5.76 μM		Ley et al. 2023
Methamphetamine	0.22 μM^1^	1 mM^2^	^1^Schepers et al. 2003 ^2^Cohen 1975
Norephedrine	0.50 μM		Toennes et al. 2003
Pseudoephedrine	1.33 μM		Flanagan et al. 2018

In the first column the drugs of interest are listed. In the second column, the plasma levels in micromolar (10-6 M) concentrations are given. This is meant to give an estimate what peak plasma concentrations are needed to normally elicit a clear somatic or psychological effect in the drug user at drug concentrations usually employed in the market. In the third column, some toxic drug concentrations are listed: here severe somatic or psychiatric side effects led to the entrance of the user to the hospital. Fourth column gives references

In isolated mouse cardiac preparations, cathine slightly increased the force of contraction; this positive inotropic effect was increased by a phosphodiesterase inhibitor (Hußler et al. 2023, Table 6). As with other drugs of interest, cathine was more effective in increasing the force of contraction in isolated human right atrial preparations. These positive inotropic effects were attenuated by propranolol and cocaine (Hußler et al. 2023, Table 6). Hence, the positive inotropic effects of cathine in the isolated human atrium and extension in the human ventricle are mediated mainly via β-adrenoceptors (Hußler et al. 2023, Neumann et al. 2023d). Moreover, this would mean that the cardiac effects of cathine in patients should be reduced by propranolol or other β-adrenoceptor antagonists. However, this is currently only a hypothesis. As mentioned above, α-adrenoceptors antagonists should perhaps be added to β-adrenoceptor antagonists to impede coronary constriction under these conditions.

Cathine is reported to increase the heart’s beating rate (Table 4) in, for example rabbits (Kalix 1983). This effect is likely mediated by noradrenaline or dopamine release, not by serotonin, because rabbits do not express 5-HT_4_-serotonin receptors in the heart (Neumann et al. 2017, 2023a, b, c, d). Data in isolated mouse right atrial preparations detect a small positive chronotropic effect of cathine (Hußler et al. 2023). Thus, one could postulate that cathine might exert a positive chronotropic effect by releasing noradrenaline locally in sinus node cells in humans and stimulating human β-adrenoceptors in the sinus node pacemaker cells. However, this needs to be studied further in human tissues or cells. This can be problematic in patients suffering from angina pectoris, which worsens when the heart rate increases as perfusion of coronaries occurs in diastole, and diastole is shortened when the heart rate increases.

**Table 4:** Cardiac and non-cardiac side effects of the drugs of interest

	Cardiac side effects	Non-cardiac side effects	References
Amphetamine	Vasoconstriction^3^, hypertension^1^, tachycardia^1^, other arrhythmias^1^	Hallucinations^2^	^1^Goodman and Gilman 2023, ^2^Shoptaw et al. 2009, ^3^Liles et al. 2007
Cathine: D-norpseudoephedrine	Vasoconstriction, heart failure, hypertension, tachycardia	Oral infection, paradontosis, hallucinations	El-Menyar et al. 2015
Cathinone	Tachycardia^1^, hypertension^1^	Hallucinations^2^	^2^Boroda and Akhter 2008, ^1^Bolli 2008
DOI	Hypotension^1^, bradycardia^1^	Hallucinations^2^	^1^Dedeoglu and Fisher 1991 ^2^Shulgin et al. 2011
DOM	Hypotension, bradycardia	Hallucinations	Snyder et al. 1968
Ephedrine	Hypertension, heart rate	Hallucinations	Boroda and Akhter 2008
L-Norpseudoephedrine	Tachycardia, hypertension	Hallucinations	Dunlop et al. 2018
MDMA: 3,4-methylene-dioxymethamphetamine	^1^Tachycardia, ^1^hypertension	Hallucinations^2^	^1^Holze et al. 2020, ^2^Shulgin et al. 2011
Mephedrone: 4-methyl-methcathinone	Tachycardia^1^, hypertension^1^	Hallucinations^2^	^1^Varner et al. 2013 ^2^Wood et al. 2011, ^3^Regan et al. 2011
Mescaline	Bradycardia^1^ hypotension^1^	Hallucinations^2^	Orzechowski and Goldstein 1973, ^2^Boroda and Akhter 2008,
Methamphetamine	Hypertension^3^, cardiac failure^3^, stroke^3^, myocardial infarction^3^, arrhythmias^1^	Psychosis^2^, schizophrenia^2^, depression^2^, dependence^2^	^1^Kaye et al. 2007, ^1^Derlet and Horowitz 1995, ^2^Yang et al. 2018, ^3^Ho et al. 2009, ^3^Huang et al. 2016; ^3^Lappin et al. 2017; ^3^Wijetunga et al. 2003; ^3^Zamanian et al. 2018; ^3^Zhao et al. 2018
Norephedrine	Hypertension^1^	Hallucination^2^	^1^Moya-Huff and Maher TJ 1987, ^2^Escobar and Karno 1982
Pseudoephedrine	Tachycardia^1,2^, hypertension^1,2^	Hallucination^3^	^1^Bolli 2008, ^2^Drew et al. 1978, ^3^Green et al. 2017

In the first column the drugs of interest are listed. Second column gives common cardiac side effects in humans (or animals if human data are lacking) and fourth column other human side effects. Fourth columns give the references.

Currently, no clinical studies on cathine are on record (Table 2), and no clinical indications seem to exist (Table 5). In the past, cathine was used as a nasal decongestant and as an anorectic preparation (Keup 1986).

**Table 5:** Indications and WADA-prohibition of the drugs of interest

	Indications	Doping drug	Reference
Amphetamine	Obesity, norcolepsy, attention-deficit disorders		Docherty 2008
Cathine: D-norpseudoephedrine			Docherty 2008
Cathinone			Docherty 2008
DOI			WADA 2021
DOM			WADA 2021
Ephedrine		1,2	^1^Docherty 2008 ^2^Shekelle et al. 2003 Miller 2004
L-Norpseudoephedrine			WADA 2021
MDMA: 3,4-methylene-dioxymethamphetamine			Docherty 2008
Mephedrone: 4-methyl-methcathinone			WADA 2021
Mescaline			WADA 2021
Methamphetamine			WADA 2021
Norephedrine			Docherty 2008
Pseudoephedrine			WADA 2021

In the first column the drugs of interest are listed. Second column lists common clinical indications for these drugs. Third column gives whether this drug is used in athletes for doping purposes and is this prohibited. Fourth column gives references. In some cases, it is debatable to what extent the doping agencies really prohibit this drug and expert opinion should be consulted

### Cathinone

Cathinone (benzoylethanamine) is an oxidation product of cathine (Fig. 1a) and is considered an amphetamine-related compound (β-ketone amphetamine: Simmons et al. 2018). Cathinone has one chiral carbon atom (Fig. 1a, Table 1) and thus gives rise to two enantiomers, S-cathinone and R-cathinone (Rothman et al. 2003). Cathinone is like cathine, formed naturally in the plant Catha edulis from phenylalanine (Hagel et al. 2011, Table 1). Cathinone is thought to be the active ingredient of Catha edulis (Friebel and Brilla 1963, Kalix 1983) and induces typical mood improvement attributed to chewing leaves of the Catha edulis plant (Kalix 1983). In mediaeval Arabic medicine, khat was used to treat depression and suppress feelings of hunger in travellers (Kalix 1996). Cathinone may cause hallucinations (Balint et al. 2009).

There are reports that pure cathinone is sometimes used as a recreational drug orally or by injection (Simmons et al. 2018). However, this is infrequent. One has speculated that users may shy away from using pure cathinone because it is chemically unstable and, upon storage, rapidly loses its hallucinogenic usefulness (Simmons et al. 2018).

Perorally applied cathinone is in part metabolised to cathine. More specifically, cathinone can be metabolised to 93% (reduced) in humans using CYP2D6 to L-norpseudoephedrine and cathine (Fig. 1a, Brenneisen et al. 1986, Silva et al. 2022). Therefore, one could argue that L-norpseudoephedrine and D-norpseudoephedrine (cathine, the primary metabolite) act as active metabolites to prolong the duration of action of cathinone in vivo. Inhibitors of CYP2D6 are expected to prolong the presence of cathinone in plasma (Silva et al. 2022).

Cathinone is transported by OCT2 and OCT3 through the brain and cardiac membranes (Table 8). Thus, inhibitors of these proteins should attenuate the effects of cathinone in the brain and heart. Moreover, cathinone can inhibit MAO activity (Nencini et al. 1984). Hence, adding MAO inhibitors such as selegiline (Fig. 1a) should prolong the action of cathinone in the heart.

The S-cathinone could release serotonin, dopamine and noradrenaline from rat brain preparations (Rothman et al. 2003, Table 7). S-Cathinone is more potent in releasing noradrenaline than serotonin or dopamine (Rothman et al. 2003). Cathinone reduces the phosphorylation state of transcription factors in human leucocytes (Bredholt et al. 2013). These dephosphorylations were quantitatively and qualitatively different from those induced by cathine (Bredholt et al. 2013). This argues for slight differences in the mechanism of action of cathine and cathinone in the human body and, therefore, conceivably also in the human heart. The clinical effects could be explained by khat-induced increased levels of serotonin and noradrenaline in brain structures.

Cathinone can increase the heartbeat in many animals, such as dogs, rabbits and guinea pigs (Kalix 1983, Table 5). In anaesthetised dogs, cathinone increases the heart rate (Kohli and Goldberg 1982). Similarly, in anaesthetised rats, cathinone had an indirect mechanism via the release of noradrenaline to increase the heart rate (Alsufyani and Docherty 2015).

In isolated right and left atrial preparations from guinea pigs, cathinone increased the force of contraction and beating rate with a half maximum effective concentration of about 1 μM (Gugelmann et al. 1985). These contractile effects of cathinone were missing when guinea pigs were pre-treated with reserpine to empty noradrenaline stores in the heart (Gugelmann et al. 1985). These findings in guinea pig cardiac preparations suggest that the release of noradrenaline mediated the effects of cathinone (Table 6).

**Table 6:** Cardiac effects in animals and humans in vivo and in vitro of the drugs of interest

	Animal studies	Human studies	References
Amphetamine	Mouse: PIE^3^, PCE^4^	PIE^4^, tachycardia^2^, hypertension^1,2^	^1^Holze et al. 2020 ^2^Hennissen et al. 2017 ^3^Hußler et al. 2023 ^4^Neumann et al. 2023d
Cathine: D-norpseudoephedrine	Mouse: PIE^2^, PCE^3^ rabbits: tachycardia^1^	PIE^3^	^1^Kalix 1984 ^2^Hußler et al. 2023 ^3^Neumann et al. 2023d
Cathinone	Chicken: decrease in force of contraction^4^ Guinea pigs, rats: increase in force of contraction^2,3^ Tachycardia: rabbits, dogs, rats, guinea pigs^1,5^ Mouse: PIE^6^, PCE^7^	PIE^7^	^1^Kalix 1983 ^2^Gugelmann et al. 1985 ^3^Cleary et al.^2002^ ^4^Maitai 1981 ^5^Kohli and Goldberg 1982 ^6^Hußler et al. 2023 ^7^Neumann et al. 2023d
DOI	Mouse: NIE^2^, NCE^2^, reduction in blood pressure^1^	NIE^2^	^1^Dedeoglu and Fisher 1991 ^2^Gergs et al. 2024
DOM	Mouse: NIE^3^, NCE^3^ reduced peripheral resistance^1^, reflex bradycardia^1^, reduced cardiac output^1^	Tachycardia^2^, increase in blood pressure^2^, NIE^3^	^1^Huang and Beng 1972 ^2^Synder et al. 1967 ^3^Gergs et al. 2024
Ephedrine	Mouse: PIE^2^, PCE^2^	PIE^2^, increased heart rate^1^	^1^Haller et al. 2004 ^2^Neumann et al. 2023b
L-Norpseudoephedrine	Mouse: PIE^2^, PCE^2^	PIE^2^, tachycardia^1^	^1^Haller et al. 2002, ^2^Neumann et al. 2023d
MDMA: 3,4-methylene-dioxymethamphetamine	Mouse: PIE^4^, PCE^4^	PIE^4^, increase in heart rate and blood pressure^1,2,3^	^1^Liechti and Vollenweider 2000, ^2^Lester et al. 2000, ^3^Vollenweider et al. 1998 ^4^Neumann et al. 2023b
Mephedrone: 4-methyl-methcathinone	Mouse: PIE^2^, PCE^2^, increase in heart rate and blood pressure in rats^1^	^2^PIE	^1^Varner et al. 2013 ^2^Gergs et al. 2024
Mescaline	Mouse: no PIE^2^, no PCE^2^, increase in force, reduction in beating rate in rat^1^	^2^No PIE	^1^Siegel and Orzechowski 1977 ^2^Neumann et al. 2023b
Methamphetamine	Mouse: PIE^5^, PCE^5^ neonatal rat cardiomyocytes: increase in beating rate^1^ Adult isolated perfused mouse heart negative inotropic^2^: bullfrog: increase in force^3^	PIE^5^, atrial arrhythmias^4^, ventricular arrhythmias^4^, myocardial infarction^4^, coronary vasospasm^4^, hypertension^4^, tachycardia^4^, cardiomyopathy^4^	^1^Sugimoto et al. 2009 ^2^Turdi et al. 2009 ^3^Urabe 1982 ^4^ Dominic et al. 2022 ^5^Neumann et al. 2023c
Norephedrine	Mouse: PIE, PCE	PIE	Neumann et al. 2023b
Pseudoephedrine	Mouse: PIE^2^, PCE^2^ increase in sinus rate in rat^1^	PIE^3^	^1^Kobayashi et al. 2003 ^2^Hußler et al. 2023 ^3^Neumann et al. 2023d

In the first column the drugs of interest are listed. Second column gives cardiac effects in general in animals in vivo or in vitro. Third column gives cardiac effects in general in humans in vivo or in vitro. Fourth column lists references. *PIE*, positive inotropic effect; *PCE*, positive chronotropic effect; *NIE*, negative inotropic effect; *NCE*, negative chronotropic effect

In rat cardiac ventricular preparations, cathinone potentiated the positive inotropic effects of noradrenaline (Cleary et al. 2002). This was explained by the observation that cathinone could inhibit the noradrenaline transporter in vitro (Tables 7 and 8). In chicken hearts, cathinone has a negative inotropic effect (Maitai 1981). The difference in chicken hearts (non-mammalian) compared to mammalian hearts can be explained by species differences. Cathinone is nearly unable to increase the force of contraction in mouse left atrial preparations (Hußler et al. 2023).

**Table 7:** Mechanism(s) of action of the drugs of interest

	Main cardiac mechanism	Possible additional cardiac mechanism	References
Amphetamine	Release of noradrenaline^1^	Inhibition of NET^2^, DAT^2^, VMAT2^2^, MAO^2^, SERT^2^	^1^Liles et al. 2007 ^2^Faraone 2018
Cathine: D-norpseudoephedrine	Release of noradrenaline^2^	transported by OCT2^1^ inhibition of NET^1^ and DAT^1^	^1^Jensen et al. 2021 ^2^Hußler et al. 2023
Cathinone	Release of noradrenaline^1,2,3^	transported by OCT3^4^ and OCT4^4^	^1^Kalix 1984 ^2^Gugelmann et al. 1985 ^3^Alsufyani and Docherty 2015 4 Jensen et al. 2021
DOI	Brain mediated^3^	binds to β_2_^1^-, α_2_^1^-, 5HT_2A,B_-receptors^1,2^	^1^Ray 2010 ^2^Titeler 1988 ^3^Dedeoglu and Fisher 1991
DOM	Brain mediated		Tadepalli et al. 1975
Ephedrine	Release of noradrenaline and serotonin^1^	Histamine receptor agonist^3^, α_2_- and β-adrenoceptor- agonist^2^	^1^Rothman et al. 2003 ^2^Vansal and Feller 1999 ^3^Kawasuji et al. 1996
L-Norpseudoephedrine	Release of noradrenaline^1^		^1^Hußler et al. 2023
MDMA: 3,4-methylene-dioxymethamphetamine	Release of noradrenaline^1^	α_1,2_- and β-Adrenoceptor-, 5-HT_2A_-serotonin agonist^1,2^	^1^Docherty 2008 ^2^Battaglia et al. 1988
Mephedrone: 4-methyl-methcathinone	Release of noradrenaline^1^	Binding to 5-HT_2A_-serotonin and D_2_-dopamine receptors^1^	^1^Lopez-Arnau et al. 2012
Mescaline	Release of histamine^2^	Transported by OCT1^3^ 5-HT_2A_- serotonin agonist^1^	^1^Rickli et al. 2016 ^2^Orzechowski and Goldstein 1973 ^3^Jensen et al. 2021
Methamphetamine	Release of noradrenaline^1^	Transported by OCT2^2^ β-adrenoceptor antagonist^1^	^1^Turdi et al. 2009 ^2^Jensen et al. 2021
Norephedrine	Release of noradrenaline^1^	β-Adrenoceptor agonist^1^ β-Adrenoceptor antagonist^2^	^1^Vansal and Feller 1999 ^2^Urabe 1982
Pseudoephedrine	Release of noradrenaline^1^	β-Adrenoceptor agonist^1^	^1^Vansal and Feller 1999

In the first column the drugs of interest are listed. Second column lists the likely mechanism of the cardiac effect in humans based on current data in animal and human studies. Third column list ancillary mechanism of these drugs. Fourth column lists references. *NET*, noradrenaline transporter; *DAT*, dopamine transporter; *VMAT2*, vesicular monoamine transporter 2; MAO, monoamine oxidase; *SERT*, serotonin transporter; *OCT*, organic cation transporter

**Table 8:** Transporters for the drugs of interest

	Transported by	Inhibitory action on	References
Amphetamine	OCT2^2^	MAO inhibition^1^ COMT inhibition^1^ DAT inhibition^1^ VMAT2^3^ NET^3^ SERT^4^	^1^Kahlig and Galli 2003 ^2^Jensen et al. 2021 ^3^Faraone 2018 ^4^Kristensen et al. 2011
Cathine: D-norpseudoephedrine	DAT NET OCT2		Jensen et al. 2021
Cathinone	OCT2 OCT3	SERT inhibition NET inhibition DAT inhibition	Luethi and Liechti 2020
DOI		P-glycoprotein	Meyer et al. 2013b
DOM	OCT1 OCT2		Meyer et al. 2013b
Ephedrine	OCT2^2^	DAT inhibition^1^ SERT inhibition^1^ NET inhibition^1^	^1^Luethi and Liechti 2020, ^2^Jensen et al. 2021
L-Norpseudoephedrine			
MDMA: 3,4-methylene-dioxymethamphetamine		SERT inhibition NET inhibition DAT inhibition	Luethi and Liechti 2020
Mephedrone: 4-methyl-methcathinone		DAT inhibition SERT inhibition NET inhibition VMAT2 inhibition	Lopez-Arnau et al. 2012
Mescaline	OCT1, OCT2, OCT3		Jensen et al. 2021
Methamphetamine	OCT2^3^	VMAT2-inhibition^1,2^ MAO-inhibition^2^ CYP2D6^2^-inhibition^2^, noradrenaline transporter inhibition^2^	^1^Meyer et al. 2013a, ^2^Fleckenstein et al. 2007 ^3^Jensen et al. 2021
Norephedrine		DAT inhibition^1^ NET inhibition^1^ MAO inhibition^2^	^1^Rothman et al. 2003, ^2^Yu 1986
Pseudoephedrine		MAO inhibition	Ulus et al. 2000

In the first column the drugs of interest are listed. Second column lists the likely mechanism of the cardiac effect in humans based on current data in animal and human studies. Third column list ancillary mechanism of these drugs. Fourth column lists references. *NET*, noradrenaline transporter; *DAT*, dopamine transporter; *VMAT2*, vesicular monoamine transporter 2; *MAO*, monoamine oxidase; *SERT*, serotonin transporter; *OCT*, organic cation transporter; *COMT*, catechol-O-methyltransferase; CYP2D6, isoform of oxidative enzymes of the cytochrome 450 family

Cardiac side effects of khat chewing in humans have been well documented (Kalix 1992, Table 4). These included increased heart rate and blood pressure. In khat users, there seems to be an increased likelihood of myocardial infarction and cardiac arrhythmias (Silva et al. 2022). In healthy humans, applying cathinone at the usual dosage found in khat led to peak plasma levels of around 0.5 μM of cathinone (Brenneisen et al. 1990). This was accompanied by tachycardia and elevated blood pressure (Brenneisen et al. 1990). It is tempting to speculate that a release of serotonin and dopamine might also occur in the human heart, but this question has not yet been addressed. Cathinone at an oral dose of 0.5 mg/kg increases blood pressure and heart rate in humans (Brenneisen et al. 1990, Table 6). When chewing the usual amounts of khat, plasma concentrations of about 0.7 μM of cathinone were observed (Widler et al. 1994, Table 3).

Cathinone, starting at 1 μM, increased the force of contraction in isolated human right atrial preparations (Hußler et al. 2023). These effects in human isolated atrium were blocked by cocaine or propranolol (Hußler et al. 2023). Thus, they likely resulted from the indirect sympathomimetic action of cathine in the human heart; noradrenaline is released, increasing the force of contraction. In mouse right atrial preparations, we detected a small positive chronotropic effect of cathinone (Neumann et al. 2023a, b, c, d). Thus, one could postulate that cathinone might exert a positive chronotropic effect by releasing noradrenaline locally in sinus node cells in humans, but this needs to be studied.

Because of its amphetamine-like action (e.g. on cardiac performance), cathinone is found on the WADA doping list (Docherty 2008, WADA, Table 5). Currently, no clinical studies on cathinone are on record (Table 2), and no clinical indications seem to exist (Table 5).

## DOI (2,5-dimethoxy-4-iodoamphetamine)

DOI is a potent hallucinogenic drug in humans (Sadzot et al. 1989). DOI has one chiral carbon atom, similar to amphetamine (Fig. 1, Table 1). Therefore, the enantiomers R-DOI and S-DOI can be distinguished. For recreational purposes, DOI was synthesised by clandestine laboratories and self-tested in 1963 (Shulgin et al. 2011) or 1973 (Coutts and Malicky 1973, Table 1). R-DOI is more potent than S-DOI as an agonist for recombinant 5-HT_2A_-serotonin receptors (Canal and Morgan 2012). This stimulation of 5-HT_2A_-serotonin is thought to underlie the hallucinogenic effect of DOI (Canal and Morgan 2012). Besides its affinity for serotonin receptors, DOI has an affinity for several adrenergic receptors in ligand-binding assays (Ray 2010). Interestingly, the affinity of DOI at these receptors is similar, if not higher, than that of 5-HT receptors. For instance, the Ki-values (equilibrium dissociation constant, concentration at which the test ligand displaces 50% of the radioactive ligand) were reported for β_2_-adrenoceptors as 140 nM, for α_2A_-adrenoceptors as 74 nM, for α_2B_-adrenoceptors as 340 nM, for 5-HT_2A_-serotonin receptors as 165 nM, for 5-HT_2B_-serotonin receptors as 336 nM and for 5-HT_2C_-serotonin receptors as 46 nM (Ray 2010, Table 7). Only β_2_-adrenoceptors mediate a positive inotropic effect in the human heart from these receptors.

Clinically, it may be relevant that some isoforms of serotonin receptors and α_2_-adrenoceptors can directly constrict human coronary arteries (Goodman and Gilman 2023). Stimulation of central α_2_-adrenoceptors by DOI (another example would be the anti-hypertonic drug clonidine) should reduce blood pressure. In anaesthetised cats, intravenous DOI increased blood pressure but transiently reduced heart rate (McCall and Harris 1988). These effects were blocked by ketanserin, an antagonist at 5-HT_2_-serotonin receptors, and were accompanied and possibly caused by increased central activation of cardiac sympathetic nervous outflow (McCall and Harris 1988). In rats, DOI reduced peripheral resistance and thereby reduced blood pressure. This probably led reflectively to a reduced heart rate, which might explain the measured reduction in cardiac output after DOI application (Dedeoğlu and Fisher 1991, Chaouche-Teyara et al. 1994). These cardiovascular effects of DOI were explained as being mainly centrally mediated (Dedeoğlu and Fisher 1991, Chaouche-Teyara et al. 1994). However, in guinea pig papillary muscles (in the presence of prazosin, atenolol and atropine), DOI (10 μM) shortened the duration of monophasic action potentials (Le Grand et al. 1995). One would predict that this should lead to a reduction of the force of contraction, but that was not measured. Moreover, an increased propensity for cardiac arrhythmias might be predicted, but this has not yet been studied.

In isolated mouse left atrial preparations, DOI has little inotropic effect. In isolated spontaneously beating mouse right atrial preparations, DOI exerts a negative chronotropic effect. In isolated electrically stimulated human atrial preparations, DOI concentration dependently reduced the force of contraction (Gergs et al. 2024).

At least in rats, DOI induced hyperthermia via 5-HT_2A_ serotonin receptors (Mazzola-Pomietto et al. 1995). If DOI leads to hyperthermia in humans, cardiac hyperthermia alone could induce arrhythmias.

DOI is not on the WADA doping list (Docherty 2008, WADA, Table 5), probably because it does not release noradrenaline and cannot affect cardiac β-adrenoceptors. Hence, DOI cannot increase performance in athletes. Currently, no clinical studies of DOI are on record (Table 2), and no clinical indications of DOI seem to exist (Table 5).

## DOM (2,5-dimethoxy-4-methylamphetamine)

DOM is a potent hallucinogenic drug in humans (Snyder et al. 1967, Halberstadt and Geyer 2011). DOM is less potent in humans than DOI to produce hallucinogenic effects (Sadzot et al. 1989). DOM was produced clandestinely in California, USA, rapidly becoming popular in youth culture. The law enforcement agencies seized DOM (street name at the time “STP”: “Serenity, Tranquillity, Peace”). STP taken from street vendors was analysed in government analytical chemical laboratories and identified as DOM (2,5-dimethoxy-4-methylamphetamine). Hallucinogenic effects of DOM were clearly shown in government-funded studies in the USA (Snyder et al. 1968). DOM was about 10 times more potent than the hallucinogenic drug lysergic acid diethylamide (LSD) and about 100 times more potent than mescaline (see below) in inducing hallucinations in healthy humans (Snyder et al. 1968).

DOM never entered the clinic, but is still often used for basic research in biochemical studies and animal behavioural studies because it is a potent and effective drug at brain serotonin receptors (notably as a partial agonist at 5-HT_2A_-serotonin receptors: Sanders-Bush et al. 1988). Like DOI, DOM also reduces peripheral vascular resistance in rats and cats, reducing blood pressure and heart rate. The reduced heart rate might explain the measured reduction in cardiac output (Huang and Beng 1972, Tadepalli et al. 1975). As with DOI, the cardiovascular effects of DOM were explained as being mainly centrally mediated (Tadepalli et al. 1975). Significantly, DOM increases blood pressure and beating rate in humans (Snyder et al. 1967). One would predict that the increase in the beating rate induced by DOM should lead to an increase in the force of contraction (due to the positive force-frequency relationship in the human ventricle) and an increased propensity to develop cardiac arrhythmias. However, this has not yet been studied. In isolated electrically stimulated human atrial preparations, DOM concentration dependently reduced the force of contraction. This effect was not attenuated by atropine, and thus, it was not mediated by the stimulation of muscarinic receptors (Gergs et al. 2024).

It is plausible that DOM has no positive inotropic effect. DOM directly stimulates 5-HT_2_-serotonin receptors independent of any intermediate (Sanders-Bush et al. 1988). In contrast, DOM does not stimulate 5-HT_4_ serotonin receptors because we measured no positive inotropic effect of DOM in human atrial preparations (Gergs et al. 2024). DOM does not release 5-HT, dopamine or noradrenaline in the human atrium because it does not increase the force of contraction in human preparations (Gergs et al. 2024). This result is expected from released noradrenaline, dopamine or serotonin alone or in concert. One would predict that if DOM is consumed over a prolonged period, cardiac 5-HT_2B_ serotonin receptors might be stimulated, leading to a proliferation of interstitial cells in cardiac valves, their insufficiency, and finally, heart failure (discussed in: Kaumann and Levy 2006). This has not yet been reported.

If DOM stimulates 5-HT_2A_ serotonin receptors in human coronaries, the contraction of coronaries and ischaemia are expected. In isolated ovine umbilical veins, DOM exerted concentration-dependent vasoconstrictory effects and was 3.4 times more potent but less effective than serotonin (Zhang and Dyer 1990). This might manifest itself as angina pectoris, myocardial infarction or arrhythmias. This hypothesis has never been studied. Unlike DOI, DOM has not been studied on a wide panel of G-protein-coupled receptors, which would facilitate understanding the mechanism of DOM in more detail. In rats, DOM induced hyperthermia via 5-HT_5A_ serotonin receptors (Aulakh et al. 1994). If DOM leads to hyperthermia in humans, cardiac hyperthermia alone could induce arrhythmias (Lenhardt et al. 1996).

Like DOI, DOM is not found on the WADA doping list (Docherty 2008, WADA, Table 5), probably because it does not release noradrenaline and cannot affect cardiac β-adrenoceptors. Hence, DOM cannot increase performance in athletes. Currently, no clinical studies of DOM are on record (Table 2), and no clinical indications of DOM seem to exist (Table 5).

## Ephedrine

Because ephedrine exhibits two chiral carbon atoms (Fig. 1a, Table 1), four isomers are possible. They include 1R, 2R-, 1R, 2S-, 1S, 2R- and 1S,2S-ephedrine or 2-methylamino-1-phenylpropane-1-ol. Vernacular names are (+)-D-pseudoephedrine (D-ψ-ephedrine) (1R,2R), (-)-L-ephedrine (1R,2S), (+)-D-ephedrine (1S,2R) and (-)-L-pseudoephedrine (L- ψ-ephedrine) (1S,2S). Over-the-counter drug mixtures often contain racemic mixtures of ephedrine (equimolar concentrations of enantiomeric D-ephedrine and L-ephedrine) or racemic pseudoephedrine (equimolar concentrations of enantiomeric L-pseudoephedrine and D-pseudoephedrine). Nagai (1897) isolated ephedrine from the Ephedra edulis plant. Likewise, in biochemical or pharmacological experiments, racemic ephedrine or racemic pseudoephedrine are typically used; these are the products of the usual chemical synthesis reactions performed in the test tube (Table 1). Separation of enantiomers is technically possible but not regularly done for financial reasons, like most drugs used in the clinic. Biological ephedrine synthesis in the brush Ephedra sinensis has been further characterised recently and starts with phenylalanine (Morris et al. 2018).

In ligand-binding assays, ephedrine did not bind directly to α- or β-adrenoceptors (Rothman et al. 2003, Table 7). Hence, ephedrine is not a direct sympathomimetic agent, but an indirect sympathomimetic drug (Rothman et al. 2003). Fittingly, ephedrine failed to increase the myocardial cAMP content in the minced rat heart (Hull et al. 1993). In contrast, ephedrine augmented cAMP levels in cells transfected with human β-adrenoceptors receptors (Vansal and Feller 1999). This would mean that ephedrine was a direct sympathomimetic agent at these receptors in these transfected cells (Vansal and Feller 1999). One reason why ephedrine increased cAMP in transfected cells and not in rat heart could be that the expression of the adrenoceptors was much higher in transfected cells. In addition, the signal transduction of the adrenoceptors might be more effective in the transfected cell than rat cardiac tissue.

Ephedrine can release dopamine and noradrenaline, but not serotonin, in rat brain preparations (Rothman et al. 2003). However, ephedrine can release serotonin from isolated platelets (Friström et al. 1977). Hence, it is unclear whether ephedrine can release human cardiac neurotransmitters and if so which neurotransmitter.

Furthermore or alternatively, ephedrine might raise cardiac levels of noradrenaline and serotonin, because ephedrine can inhibit the activity of MAO enzymes (Ulus et al. 2000, Table 7). Ephedrine binds with low affinity to human α_2_-adrenoceptors (Rothman et al. 2003). In vivo, ephedrine can also raise histamine levels and thus indirectly stimulate cardiac histamine receptors (Kawasuji et al. 1996). In contrast to the binding data (Rothman et al. 2003), a direct stimulatory action of ephedrine on α-adrenoceptors in rat vessels or living mice was claimed to exist (Liles et al. 2006, 2007). However, these effects might be mediated by the release of endogenous noradrenaline in the vessel or the whole animal.

Ephedrine is in vitro not transported by OCT 1,3 or the dopamine-, serotonin- or noradrenaline transporters but by OCT2 (Jensen et al. 2021, Table 8). Hence, drugs that inhibit OCT2 should increase cardiac ephedrine levels and thus ephedrine action in the heart. In the USA, ephedrine was used in the 1930s orally or via inhalers to treat asthma as an alternative to adrenaline, which had to be injected (Lee 2011). When the supply of ephedrine from China failed (war between Japan and China) in the 1930s, ephedrine was synthesised in vitro (Lee 2011).

Ephedrine is still sometimes used during anaesthesia by obstetricians to raise blood pressure during childbirth (Shekelle et al. 2003, Ngan Kee and Khaw 2006, Xu et al. 2019). Therefore, ephedrine is on the WHO list of Essential Medicines (WHO 2019). Ephedrine can treat obesity, asthma and narcolepsy (Shekelle et al. 2003). Ephedrine has been used successfully in genetically based rare neurological diseases (Eirís-Puñal et al. 2020).

Ephedrine, like its isomers, can lead to hyperthermia (Arch et al. 1982) probably resulting from released noradrenaline stimulating β_3_-adrenoceptors in fat cells (Gad et al. 2021). As mentioned above, hyperthermia of the heart increases the propensity for arrhythmias. Ephedrine can increase the performance of athletes (Shekelle et al. 2003). Others noted that ephedrine is sometimes present in dietary supplements for athletes (Miller 2004) and sometimes found in dietary supplements (also called thermogenic supplements) sold for weight reduction (Haller et al. 2002). Ephedrine was placed on the list of prohibited substances of WADA (World Anti-Doping Agency - WADA 2021, Docherty 2008, Table 5).

Cardiac side effects of ephedrine include hyperthermia, tachycardia and hypertension (Boroda and Akhter 2008, Bolli 2008, Table 4). Ephedrine at high doses can lead to hallucinations in susceptible persons (Shufman et al. 1994, Boroda and Akhter 2008, Bolli 2008, Table 4). The fact that ephedrine can lead to hallucinations by stimulation of 5-HT_2A_ serotonin receptors in the brain explains the “recreational” (mis-)use of ephedrine. In some countries, ephedrine is used as a mydriatic agent (locally applied) and as a nasal decongestant in the form of drops (Miura 1887, Lee 2011). Ephedrine is present in over-the-counter drug mixtures freely available in pharmacies in some countries and these mixtures are used to reduce symptoms of the common cold. These mixtures are often taken by patients unknown to their physicians; the attending physician is sometimes surprised why these patients present with hypertension (Bolli 2008). Moreover, “ravers” use these mixtures to obtain ephedrine in nightclubs (Archer et al. 2013). Like amphetamine (vide supra), ephedrine is a metabolite of selegiline, a drug used to inhibit monoamine oxidase for the treatment of Morbus Parkinson’s disease and depression (Shin 1997, Fig. 1a). The lethal dose of ephedrine in adult humans is between 1 g and 2 g (Lee 2011). The peak plasma concentration of ephedrine at the usual dosing is 1.12 μM (Table 3).

In isolated mouse cardiac preparations, ephedrine slightly increased the force of contraction; this positive inotropic effect was increased by a phosphodiesterase inhibitor (Neumann et al. 2023b). Ephedrine was more potent and effective in increasing force in isolated human right atrial preparations than in mouse atrial preparations (Neumann et al. 2023b). These positive inotropic effects in human atrial preparations started at therapeutic drug concentrations and were attenuated by propranolol and cocaine (Neumann et al. 2023b, Table 6). Hence, the positive inotropic effects of ephedrine in the isolated human atrium and extension in the human heart are mediated mainly via β-adrenoceptors. Moreover, this would mean that the cardiac effects of ephedrine in patients should be reduced by propranolol or other β-adrenoceptor antagonists. However, this is currently only a hypothesis. Moreover, as mentioned above, antagonists at α-adrenoceptors should possibly be given simultaneously to reduce the probability of coronary occlusion by the unopposed action of noradrenaline in vascular α-adrenoceptors. The current indications of ephedrine are listed in Table 5. Currently, 92 clinical trials have tested further clinical indications of ephedrine (at clinical trials.gov, Table 2).

## MDMA

3,4-Methylene-dioxy-methamphetamine (MDMA, Fig. 1a) can cause hallucinations (Skryabin et al. 2018). It was first synthesised by the German chemist Anton Köllisch in 1912 for the Merck Company (Dunlap et al. 2018). MDMA was intended by Merck for use as an intermediate for drug production in order not to infringe patents held by the competing drug company Bayer (Table 1, Dunlap et al. 2018). At the time, MDMA was not tested in animals or humans because there was no need (Dunlap et al. 2018). In the 1950s, the United States Army started a programme to interrogate prisoners (Dunlap et al. 2018). The intention was that “truth drugs” would make prisoners more cooperative and less likely to tell lies. They tried LSD and mescaline. Based on their experiments with mescaline, they came up with the idea of testing a chemically related drug, namely MDMA (Dunlap et al. 2018). These studies were apparently not very successful and after that, MDMA was forgotten.

Around 1976, the American underground chemist Alexander Shulgin, probably by chance, performed studies on himself with MDMA. He experienced hallucinations and other mood-altering experiences (Dunlap et al. 2018) and distributed MDMA in California, USA, to younger individuals. MDMA was then tested widely and became popular in nightclubs (“ravers”, Dunlap et al. 2018). After that, MDMA was studied in medicine. In the 1970s, MDMA showed promise in the treatment of psychiatric diseases like depression, post-traumatic stress syndromes, autism disorders, some forms of schizophrenia and social anxiety (Dunlap et al. 2018). The active mechanism in MDMA probably relies on an increase in interstitial brain concentrations of dopamine, serotonin and noradrenaline. These increases in neurotransmitter concentrations result from inhibiting the function of serotonin transporters, dopamine transporters and noradrenaline transporters on the outer cell surface (Dunlap et al. 2018). Moreover, MDMA can inhibit the intracellular transport of monoamines via vesicular monoamine transporters (VMAT, Dunlap et al. 2018). MDMA also inhibits MAO enzyme activity (Dunlap et al. 2018), further elevating the concentrations of neurotransmitters. MDMA may also act as a direct receptor agonist. This has been extensively studied.

On closer examination, the potent agonistic action of MDMA is primarily on 5-HT_2A_-serotonin receptors, 5-HT_2B_- serotonin receptors, M_3_-muscarinic receptors, H_1_-histamine receptors and α_2A_-, α_2B_- and α_2C_-adrenoceptors. These receptors, with the possible exception of the H_1_-histamine receptor (review: Neumann et al. 2023c), do not increase the force of contraction or the beating rate of the human heart. Moreover, MDMA can bind to sigma 1 receptors and trace amine-associated receptors (Dunlap et al. 2018). Some of these interactions must underlie the cardiac side effects of MDMA (Dunlap et al. 2018). These side effects include cardiac arrhythmias (like tachycardia), hyperthermia (which can lead to arrhythmias), myocarditis, myocardial infarction, increased systolic blood pressure, rhabdomyolysis and the serotonin syndrome. Possibly, the side effects of illicit users are aggravated by the fact that they combined MDMA with other drugs voluntarily. Moreover, drug dealers sometimes sell their customers undeclared mixtures of drugs, typically containing in addition to MDMA also cathinone derivatives such as mephedrone (vide infra, Dunlap et al. 2018). Finally, the MDMA produced in underground laboratories shows sometimes contaminations by intermediate products or side products of the chemical synthesis. These chemical contaminations or combinations with side products may account for the neurotoxic side effects of MDMA (Dunlap et al. 2018).

The half-life of 100 mg MDMA is approximately 9 h and is prolonged if higher dosages are given (Dunlap et al. 2018). This kinetic behaviour is unusual, but well known. For example, the antiepileptic drug phenytoin also has a similar kinetic behaviour: when the dose of phenytoin is increased, the half-life of phenytoin is also increased (Goodman and Gilman 2023). This kinetic behaviour makes overdosing with MDMA especially dangerous. MDMA is metabolised in humans via the enzymes CYP2D6 and COMT (Dunlap et al. 2018). Therefore, drugs that inhibit CYP2D6 and COMT enzymes may prolong the half-life of MDMA and, therefore, its side effects. In general, MDMA can release noradrenaline but also serotonin from cells (Fig. 1b) (Rothman et al. 2003, Rickli et al. 2016).

MDMA can also inhibit MAO-A activity and thereby could raise serotonin and noradrenaline levels in the heart (Steuer et al. 2016). MDMA leads to vasoconstriction via α_1,2_-adrenoceptors and 5-HT_2A_-serotonin receptors (review: Docherty 2008). MDMA may at least bind to β-adrenoceptors (Battaglia et al. 1988); whether this is agonistic or antagonistic has not been reported. MDMA is not transported by OCT1-3 or the serotonin or noradrenaline transporters in vitro (Jensen et al. 2021). Acutely, MDMA (125 mg) in humans led to hypertension and increased heart rate, elevated body temperature and widened pupil size. These alterations were accompanied by peak MDMA concentrations of 236 ng/ml (= 1.22 μM, Holze et al. 2020). Chronic application of MDMA alters cardiac gene expression (mouse: Koczor et al. 2015). MDMA is forbidden by the WADA (World Anti-Doping Agency - WADA 2021, Docherty 2008). There have been 64 clinical trials that test possible clinical indications of MDMA (at clinicaltrials.gov, Table 2).

In the isolated mouse left atrium, MDMA exerted a positive inotropic effect that was increased by a phosphodiesterase inhibitor (Neumann et al. 2023b). In the isolated mouse right atrium, MDMA increased the beating rate, similar to the results in patients taking MDMA (Neumann et al. 2023b). In isolated human right atrial preparations, MDMA exerted a concentration-and time-dependent positive inotropic effect, which could be attenuated by propranolol or cocaine (Neumann et al. 2023b). Therefore, MDMA in the isolated human atrium and by extrapolation in the human heart in vivo acts via the release of noradrenaline. Moreover, it could be that dopamine is also involved in the cardiac action of MDMA. Dopamine probably directly stimulates β-adrenoceptors in the heart, leading to a positive inotropic effect and a positive chronotropic effect. On the other hand, these data exclude the involvement of cardiac serotonin in the inotropic effects of MDMA. Serotonin increases force in the human heart via 5-HT_4_ serotonin receptors, a process not blocked by propranolol.

### Mephedrone (4-Methyl-meth-cathinone)

Mephedrone (Fig. 1a), sometimes sold over the internet under the name “bathing salt”, was first synthesised from cathinone in 1929 (Sanchez) for purely theoretical reasons (Table 1). Mephedrone contains one chiral carbon atom (Fig. 1); thus, two enantiomers of mephedrone are known, called S- and R-mephedrone (Table 1). S-Mephedrone is unstable and rapidly converted to racemic mephedrone (Simmons et al. 2018). S-Mephedrone binds to but is no agonist but probably an antagonist at 5-HT_2A_-serotonin receptors, 5-HT_2B_-serotonin receptors or 5-HT_2C_-serotonin receptors (Philogene-Khalid et al. 2017). S-Mephedrone at 1 μM was inactive in binding to a battery of G-protein coupled receptors, namely all adrenergic and all muscarinic receptors (Philogene-Khalid et al. 2017). In contrast, mephedrone inhibits serotonin transporters, dopamine transporters, noradrenaline transporters and VMAT2 (Hadlock et al. 2011, Baumann et al. 2012, Martinez-Clemente et al. 2012). Hence, mephedrone is probably not an agonist at β-adrenoceptors.

Mephedrone is metabolised by the enzyme CYP2D6, and the active metabolite, nor-mephedrone, is formed (Mayer et al. 2016). Thus, inhibitors of CYP2D6 are expected to potentiate the cardiac side effects of mephedrone. In about 2007, mephedrone was identified in the illicit drug market (Wood et al. 2010, James et al. 2011). It has been suggested that mephedrone, like other cathinone derivatives, convinced users of its better chemical stability in storage than cathinone itself (Simmons et al. 2018). Mephedrone is generally not used alone but combined with other drugs of abuse, such as MDMA (Mead and Parrot 2020). Mephedrone can be taken orally, sniffed or injected intravenously (Mead and Parrot 2020) or intramuscularly (Wood et al. 2010).

Mephedrone increased blood pressure and heart rate in rats (Varner et al. 2013). The heart rate might have increased by stimulating β-adrenoceptors, and increased blood pressure was mediated by α-adrenoceptors (Varner et al. 2013). In spontaneously beating isolated mouse right atrial preparations, mephedrone at 10 μM exerted a positive chronotropic effect blocked by propranolol (Gergs et al. 2024). This means that mephedrone, in principle, can raise the heart rate via direct action on the sinus node. In the isolated human atrium, mephedrone (10 μM) increased the force of contraction; this effect was antagonised by 10 μM propranolol.

In humans, mephedrone can increase blood pressure (Wood et al. 2010, Regan et al. 2011). Currently, there are two clinical trials with mephedrone (www.clinicaltrials.gov, Table 2). They mainly address the pharmacokinetic of pharmacodynamic effects of mephedrone in healthy subjects and interactions with ethanol. In isolated electrically stimulated human atrial preparations, mephedrone concentration dependently increased the force of contraction; this effect was antagonised by propranolol, suggesting the release of noradrenaline (Gergs et al. 2024). The cardiac side effects of mephedrone include tachycardia and hypertension (James et al. 2011, Wood et al. 2011). Mephedrone has been claimed to cause deadly intoxication possibly due to its cardiac side effects (Loi et al. 2015).

### Mescaline

Mescaline (Fig. 1a) is an alkaloid first isolated from a particular species of peyote, a cactus grown mainly in northern Mexico (Goodman and Gilman 2023, Table 1, Fig. 1a). However, the earliest use of mescaline was likely 6000 years ago in what is now Peru (Cassels and Sáez-Briones 2018). Mescaline-containing plant extracts were used by autochthonous cultures in the Americas to induce hallucinations in social ceremonies (reviewed in Lewin 1888, 1924) and are still being used (Cassels and Sáez-Briones 2018). Mescaline directly stimulates brain 5-HT_2A_ serotonin receptors, which explains why mescaline can lead to hallucinations (Rickli et al. 2016). Mescaline binds not only to 5-HT_2A_-serotonin receptors but also to 5-HT_2C_-serotonin receptors and α_2_-adrenoceptors (Cassels and Sáez-Briones 2018).

Mescaline was identified and named by Lewin, purified and self-tested by Heffter and synthesised by Späth (Lewin 1888, Heffter 1894, Späth 1919). The cactus peyote that was first investigated and was initially called Anhalonium Lewinii after Louis Lewin (Cassels and Sáez-Briones 2018). It is currently called Lophophora williamsii (Cassels and Sáez-Briones 2018). Mescaline is also found in many species of Trichocereus all over the Americas (Cassels and Sáez-Briones 2018). The content of mescaline differs according to the species of cactus. A review of plants producing mescaline (from tyrosine) is recommended for details (Cassels and Sáez-Briones 2018). Mescaline has a plasma half-life of about 6 h (Cassels and Sáez-Briones 2018). Mescaline is seldom used outside of religious ceremonies. Mescaline is rarely used in the illicit drug market (Cassels and Sáez-Briones 2018). This may be because mescaline is not as potent as other hallucinogenic drugs. One needs at least 300 mg of pure mescaline (about 20 times more than 6 g of cactus) to experience hallucinations (self-experiment: Heffter 1894).

Mescaline is well transported by OCT1, less transported by OCT2 and OCT3, and is not transported by noradrenaline or serotonin transporters (Jensen et al. 2021). If OCT1 is genetically altered or drugs that inhibit OCT1 are co-administered, increased plasma levels and thus augmented functional effects of mescaline are expected (Koepsell 2021). Mescaline (100 μM or more) exerted positive inotropic effects in isolated rat atria but led to a negative chronotropic effect in the organ bath (Siegl and Orzechowski 1977). Mescaline can release histamine in vivo in dogs and cats (Orzechowski and Goldstein 1973). In that study, mescaline-induced bradycardia in dogs and cats in vivo. This effect was not blocked by atropine or sympathectomy and was probably not mediated by muscarinic receptors or adrenoceptors (Orzechowski and Goldstein 1973).

The negative chronotropic effect of high concentrations of mescaline (1 mM) can be attenuated by the H_2_-histamine receptor antagonist metiamide in spontaneously beating right atrial preparations from rats and was thus suggested to be H_2_-histamine receptor-mediated (Siegl and Orzechowski 1977). Four clinical trials examined the indications of mescaline (at clinicaltrials.gov, Table 2). One study did not detect either positive or negative inotropic effects in isolated mouse atria or isolated human atria (Neumann et al. 2023b). One could argue that this is consistent with a lack of mescaline to release noradrenaline in the brain. This finding does not mean that cardiac side effects of mescaline are absent, but simply that inotropy is not affected acutely in the atrium. How mescaline affects the force of contraction in the human ventricle remains to be studied. It is conceivable that chronic use of mescaline might alter gene expression in the heart and, hence, cardiac function. But, such data are apparently not found in the literature.

### Methamphetamine

Methamphetamine (N-methylamphetamine, desoxyephedrine, N-methyl-1 phenylpropane-2-amine, N,α-dimethylphenethylamine) is a phenylpropaneamine but can also be regarded as a phenylethane derivative (Fig. 1a). Methamphetamine displays structural similarities to 2-amino-1-(3,4-dihydroxyphenyl)-ethanol (noradrenaline). Methamphetamine has one chiral centre (Table 1, Fig. 1a). Hence, the enantiomers (S)-(+)-methamphetamine and (R)-(-)-methamphetamine must be distinguished. Reduction of ephedrine was the first way to obtain methamphetamine (Nagai 1897, Table 1). Synthesis from cheap phenylacetone (combining 1-phenyl-2-propanon and methylamine) was described later (German patent 1937). This synthesis by drug companies has led to the misuse of methamphetamine (Abbruscato and Trippier 2018). The base methamphetamine forms a salt with hydrochloric acid. This salt is known as “crystal meth” (Abbruscato and Trippier 2018). This salt has the pharmacokinetic advantage that it can be inhaled. Thus, effects of methamphetamine in the brain (but also in the heart) are obtained more rapidly by inhalation than by the oral application of methamphetamine.

In the street, only racemic methamphetamine is sold (Abbruscato and Trippier 2018). Methamphetamine has a half-life of 8–12 h in the plasma (Abbruscato and Trippier 2018). Methamphetamine leaves the body 93% unchanged. If metabolites of methamphetamine are carefully sought, one can find that amphetamine and norephedrine are metabolites of methamphetamine (Carvalho et al. 2012, Goodman and Gilman: 2023). Thus, amphetamine and norephedrine can be regarded as active metabolites of methamphetamine (vide supra and vide infra). Methamphetamine can be taken via the oral route, the intravenous route, the intranasal route and via inhalation (Abbruscato and Trippier 2018). Methamphetamine is the primary metabolite of selegiline (Fig. 1a), a drug that inhibits MAO for the treatment of Morbus Parkinson and depression (Shin 1997).

S-Methamphetamine is only illicitly used by addicts. D-Methamphetamine is present in some decongestants (Abbruscato and Trippier 2018). D-Methamphetamine is allowed in some countries to treat attention-deficit disorder and obesity (Abbruscato and Trippier 2018).

In contrast to the chemically related noradrenaline (Fig. 1a), methamphetamine is viewed as an indirect sympathomimetic drug in the brain. Methamphetamine acts mainly by releasing noradrenaline from tissue or cells (Fig. 1b). Released noradrenaline then activates both α- and β-adrenoceptors. S-(+)-methamphetamine can induce the release of serotonin, dopamine and noradrenaline from rat brain preparations with increasing potency (Rothman et al. 2003). This explains the central and peripheral effects of methamphetamine. Regarding drug targets for methamphetamine, a vast number of studies can be found in the literature. For example, methamphetamine can inhibit the function of VMAT2 (Meyer et al. 2013a, b) and thus lead to the release of noradrenaline (Fig. 1b). Methamphetamine can inhibit the action of the enzymes MAO-A and MAO-B. Methamphetamine can also slow the metabolism of itself by impeding the activity of cytochrome CYP2D6. Methamphetamine can increase CREB-phosphorylation (Krasnova et al. 2016), a result of β-adrenoceptor stimulation and cAMP and can subsequently activate cAMP-dependent protein kinases (Krasnova et al. 2016; Shaerzadeh et al. 2018). Methamphetamine may also inhibit the transporters of noradrenaline in cell surface membranes (Fleckenstein et al. 2007). It is unclear whether this occurs in the human heart.

Recreational drugs, like methamphetamine, can lead to intoxications and death due to cardiac arrhythmias. For instance, coronary vessel occlusion following intake of methamphetamine in users can lead via α-adrenergic receptors to deadly cardiac arrhythmias. In such intoxications, levels of up to 1 mM of methamphetamine have been reported (Cohen 1975). Chronic application of methamphetamine can alter gene expression in the brain (Yamamoto et al. 2005).

Over decades, methamphetamine became popular for increasing wakefulness, enhancing aggressiveness, improving physical endurance and losing weight.

Methamphetamine was used under the name Pervitin® (colloquial name: “Panzerschoko-lade”, “tank chocolate”) by the German army (Wehrmacht) from 1939 to 1945 (Ohler 2016). Some claim that the German political leaders at that time were methamphetamine addicts (Ohler 2016). Methamphetamine-induced hallucinations have been firmly established, for instance, in Japan, when the US forces of occupation retreated and their methamphetamine stocks entered the Japanese market (Yui et al. 2000). Methamphetamine has been used by athletes for decades. Mountain climbers (e.g. Reinhold Messner in the Himalayas) used methamphetamine to fight hunger, sleep and fatigue (Morelli and Tognotti 2021). Thus, methamphetamine found its place on the list of drugs prohibited by WADA to impair its use in doping in professional sports.

Methamphetamine is still one of the most often used illicit central stimulants (Archer et al. 2013, Hoffmann et al. 2018). Methamphetamine can harm not only the central nervous system but also peripheral tissues. The primary central side effects of acute or chronic use of methamphetamine are psychosis, schizophrenia, depression and dependence (Yang et al. 2018). Methamphetamine also induces peripheral detrimental effects; methamphetamine in drug users may lead to cardiac arrhythmias (Kaye et al. 2007, Derlet and Horowitz 1995). Methamphetamine can also induce hypertension, resulting in fibrosis, left or right ventricular cardiac hypertrophy, cardiac failure or stroke, myocardial infarction and finally sudden cardiac death (Ho et al. 2009; Huang et al. 2016; Lappin et al. 2017; Wijetunga et al. 2003; Zamanian et al. 2018; Zhao et al. 2018). A typical peripheral side effect of methamphetamine (as with khat users vide supra) is tooth decay, likely caused by constriction of the mouth arteries (Abbruscato and Trippier 2018).

In cultured neonatal rat cardiomyocytes, methamphetamine decreased Ca^2+^ transients and augmented their spontaneous beating rate (Sugimoto et al. 2009). These data are mechanistically interesting. In cultured neonatal cardiomyocytes, if no contaminant nerve cells are present, methamphetamine cannot release noradrenaline from the nerve cells. Alternatively, methamphetamine could have released noradrenaline from cardiomyocytes. There are data that cardiomyocytes can produce noradrenaline. If this is the case, further investigation is required. In other words, one could repeat these experiments in the presence of propranolol to rule out or prove the involvement of β-adrenergic receptors.

In isolated mouse hearts, methamphetamine up to 1000 μM did not alter the beating rate, but diminished contractility (Turdi et al. 2009). This may mean that the effect of methamphetamine on the heart is age and/or species dependent.

Moreover, in human cardiac preparations, methamphetamine increases the force of contraction. This effect was blocked by propranolol and cocaine, suggesting that methamphetamine acts as an indirect sympathomimetic drug in the human heart (Neumann et al. 2023b). It is unlikely that methamphetamine directly stimulates β-adrenoceptors and thus increases the force of contraction and the beating rate. This conclusion is based on the observation that the contractile effects of methamphetamine were absent in the presence of cocaine (Neumann et al. 2023b).

Yet, nearly 400 clinical studies using methamphetamine are found at the time of this writing at www.clinicaltrials.gov, where clinical studies are archived (Table 2). These past or ongoing clinical studies try, for instance, to clarify the pharmacokinetics of methamphetamine or to test drugs to treat symptoms of methamphetamine dependence or methamphetamine withdrawal in humans (Table 2).

### Norephedrine

Norephedrine, a N-demethylated ephedrine derivative (Fig. 1a), is usually regarded as an indirect sympathomimetic agent. Norephedrine shows two enantiomers (Table 1), namely (-) norephedrine and (+) norephedrine (Table 1). (-) Norephedrine was more potent than (+) norephedrine in releasing noradrenaline and dopamine from rat brain preparations (Rothman et al. 2003, Table 7). However, neither enantiomer of norephedrine released serotonin from rat brain preparations (Rothman et al. 2003). Likewise, racemic norephedrine failed to increase the myocardial cAMP content in minced rat hearts. (Hull et al. 1993).

Norephedrine is found in the same plants as ephedrine and is thus a natural product (Table 1). Norepinephrine could increase cAMP levels in cells transfected with human β-adrenoceptors, suggesting that norephedrine binds as an agonist at these receptors (Vansal and Feller 1999, Table 7). One might speculate that because adrenoceptor was very highly overexpressed in transfected cells (more than under physiological conditions in the heart), a cAMP increase became detectable (Vansal and Feller 1999). In contrast, others have found an affinity of norephedrine for adrenoceptors (Rothman et al. 2003). Hence, the direct effects of norephedrine on adrenoceptors in the human cardiac atrium cannot be ruled out but appear controversial. In pithed rats, racemic norephedrine and its isomers increased blood pressure or beating rate. However, the (-) isomer was more effective and potent than the (+) isomer or racemic norephedrine (Moya-Huff and Maher).

Norephedrine was associated with an increased risk of haemorrhagic stroke (Table 4). In many cases, dietary supplements contain a synthetic racemic form of norephedrine called (not quite correctly based on its chemistry) “phenylpropanolamine” (FDA 2000, Watson et al. 2010). Norephedrine is still detected in illicitly marketed diet supplements for athletes (Table 2). In many drugs, racemic mixtures containing L-norephedrine and D-norephedrine are sold over the counter; racemic mixtures are cheaply synthesised through normal (non-chiral) chemical synthesis. Separating chiral compounds would be more costly; thus, this is usually not done, as with most drugs used in the clinic.

Using the search term “norephedrine”, one finds 56 clinical trials (at clinical trials.gov, Table 2). Norephedrine can inhibit MAO activity and thereby might raise levels of noradrenaline and serotonin (Yu 1986).

### L-Norpseudoephedrine

On a molar basis, L-norpseudoephedrine is more effective in raising blood pressure in living anaesthetised rats than D-norpseudoephedrine (cathine) or racemic norpseudoephedrine (Moffa-Huff et al. 1987). Like cathine, but about twofold more potent, L-norpseudoephedrine can release noradrenaline and dopamine, but not serotonin, from rat brain preparations (Rothman et al. 2003). L-Norpseudoephedrine is present in all drug preparations containing racemic norpseudoephedrine. L-Norpseudoephedrine is sometimes misused in anorectic drug preparations (Moya-Huff et al. 1987). Few pharmacological data on L-norpseudoephedrine are available. Cathinone can be metabolised (reduced) in humans via CYP2D6 (to a minor extent) to L-norpseudoephedrine (Brenneisen et al. 1986).

Therefore, one could argue that L-norpseudoephedrine, as an active metabolite, prolongs the duration of action of cathinone in vivo. Moreover, inhibitors of CYP2D6, such as cimetidine, prolong the cardiac effects of L-norpseudoephedrine. Currently, no clinical studies on L-norpseudoephedrine are on record (Table 2), and no clinical indications of L-norpseudoephedrine cathine seem to exist (Table 5).

### Pseudoephedrine

Pseudoephedrine is an ephedrine derivative (Fig. 1a) found in the same plants that produce ephedrine (Table 1). Four diastereomers of ephedrine are possible. One of the four isomers of ephedrine is pseudoephedrine, also called 1S,2S(+) pseudoephedrine or threo-ephedrine (Table 1). The enantiomer of (+) pseudoephedrine is (-) pseudoephedrine, and they exhibit pharmacological differences. (-)-Pseudoephedrine, but not (+) pseudoephedrine, can inhibit serotonin uptake in the synaptic cleft, increasing local serotonin concentrations (Rothman et al. 2003, review Docherty 2008, Table 7). Hence, one can speculate that (-) pseudoephedrine can potentiate the contractile effects of serotonin in the human heart.

Both enantiomers of pseudoephedrine can lead to the release of dopamine and noradrenaline but not to the release of serotonin from rat brain preparations (Rothman et al 2003, Table 7). However, (+) pseudoephedrine is more potent in releasing noradrenaline or dopamine than (-) pseudoephedrine. Pseudoephedrine can also inhibit the degradation of serotonin by MAO-A (Ulus et al. 2000, Table 8), which is also expected to lead to the potentiated effects of serotonin in the heart. Pseudoephedrine has been used for decades as a nasal decongestant and in obscure mixtures to treat the common cold or relieve the nasal congestion of viral infections (Table 2). This reduction in nasal congestion using pseudoephedrine is attributed to vasoconstriction in the nose blood vessels via the stimulation of nasal vascular α_2_-adrenoceptors. However, in biochemical studies with recombinant receptors in transfected cells, (-) pseudoephrine or (+) pseudoephrine up to 10 μM did not functionally stimulate these receptors (Rothman et al. 2003).

Based on these data, pseudoephedrines are unlikely to act as direct stimulators of intranasal α_2_-adrenoceptors on vessels. Pseudoephedrine is almost certainly not an antiviral agent. Pseudoephedrine can have severe systemic side effects like hypertension, and thus, its use to treat viral infections can do more harm than good. Sometimes pseudoephedrine is used without prescription in liquids, solids or ointment to treat the common cold (Głowacka and Wiela-Hojeńska 2021). Intravenous pseudoephedrine is used in recreational users (Sullivan 1996). Nose drops containing phenylpropanolamine can cause hallucinations (Escobar Karno 1982, Sauder et al. 1997).

In isolated mouse cardiac preparations, pseudoephedrine slightly increased the force of contraction; this positive inotropic effect was increased by a phosphodiesterase inhibitor (Hußler et al. 2023). Like other drugs of interest, pseudoephedrine was more effective in increasing force in isolated human right atrial preparations than in mouse atrial preparations. These positive inotropic effects were attenuated by propranolol and cocaine (Hußler et al. 2023, Neumann et al. 2023d, Table 6). Hence, the positive inotropic effects of pseudoephedrine in the isolated human atrium and, by extension, the human heart are mediated primarily via β-adrenoceptors. Moreover, this would mean that the cardiac effects of pseudoephedrine in patients could be reduced by propranolol or other β-adrenoceptor antagonists. However, this is currently only a hypothesis. Pseudoephedrine is used as a doping agent in sports and is forbidden by WADA.

### Summary and outlook

Some drugs of interest can release noradrenaline, dopamine or serotonin from brain preparations. If these drugs also release monoamines in the heart, this could be the mechanism for the cardiac effects of these drugs. The situation is clear for noradrenaline: it acts in the heart via adrenoceptors. More specifically, noradrenaline could increase the force of contraction in the human atrium and ventricle via β-adrenoceptors or α-adrenoceptors. Noradrenaline via α1-adrenoceptors could lead to harmful constriction of the coronary arteries, leading to cardiac arrhythmias. Stimulation of β-adrenoceptors in the sinus node of humans could increase the beating rate and exacerbate pre-existing angina pectoris. Moreover, β-adrenoceptor stimulation can induce arrhythmias.

All drugs of interest stimulate brain 5-HT_2_- serotonin receptors. None seems to stimulate human cardiac 5-HT_4_-receptors. Moreover, for the current drugs of interest three groups can be formed. The first group has no direct cardiac effect in the human atrium: mescaline. The second group exerts by uncertain mechanism (possibly via antagonism at adrenergic receptors) a negative inotropic effect in the human atrium: DOI and DOM. The remainder of the drugs of interest release in the human atrium noradrenaline: this noradrenaline mediates their cardiac effects. Currently, it is unknown whether and to what clinical relevance the drugs of interest activate or block additional cardiac receptors.

Serotonin can increase the force of contraction in the human atrium and a failing human ventricle via 5-HT_4_-serotonin receptors. Moreover, serotonin can induce supraventricular arrhythmias in the human heart. Less clear is the cardiac role of dopamine (Neumann et al. 2023a). Lower concentrations of cardiac dopamine could stimulate β-adrenoceptors with possible complications. Dopamine might also stimulate cardiac dopamine receptors, leading to arrhythmias (Neumann et al. 2023a).

We hypothesise that the cardiac action of drugs of interest, except mescaline, involves hitherto unknown mediators and mechanisms. This will become apparent only once we better understand the physiology of the healthy and the failing heart. It is also not farfetched to predict that the side effects of indirect sympathomimetic agents in the heart will depend on monoamines in the food, additional drugs (e.g. antidepressant MAO enzyme inhibitors) and pre-existing health conditions. Nevertheless, the current antidepressant and antipsychotic agents have detrimental cardiac side effects. Carefully controlled studies of some indirect sympathomimetic but hallucinogenic drugs in psychiatric diseases should be encouraged.

## Abbreviations

ADHD: Attention-deficit and hyperactivity disorders (ADHD)
cAMP: 3´,5´-Cyclic adenosine monophosphate
CREB: cAMP responsive element, a transcription factor
CYP2D6: Isoenzyme of cytochrome P450
CYP3A4: Isoenzyme of cytochrome P450
DAT: Dopamine transporting proteins
DOI: 2,5-Dimethoxy-4-iodoamphetamine
DOM: 2,5-Dimethoxy-4-methylamphetamine
Ki-values: Molar equilibrium dissociation constant of ligand at receptor
LSD: Lysergic acid diethylamide
μM: Micromole per litre
nM: Nanomole per litre
mM: Millimole per litre
MAO: Monoamine oxidases
MDMA: 3,4-Methylene-dioxymethamphetamine
NET: Noradrenaline transporting proteins
OCT: Organic cation transporter
SERT: Serotonin transporter
VMAT2: Vesicular monoamine transporter
WADA: World Anti-Doping Agency
WHO: World Health Organization
